# Enhanced Microvasculature Formation and Patterning in iPSC–Derived Kidney Organoids Cultured in Physiological Hypoxia

**DOI:** 10.3389/fbioe.2022.860138

**Published:** 2022-06-13

**Authors:** Anika Schumacher, Nadia Roumans, Timo Rademakers, Virginie Joris, Maria José Eischen-Loges, Martijn van Griensven, Vanessa L.S. LaPointe

**Affiliations:** ^1^ Department of Cell Biology–Inspired Tissue Engineering, MERLN Institute for Technology-Inspired Regenerative Medicine, Maastricht University, Maastricht, Netherlands; ^2^ Department of Instructive Biomaterials Engineering, MERLN Institute for Technology-Inspired Regenerative Medicine, Maastricht University, Maastricht, Netherlands

**Keywords:** kidney organoid, hypoxia, VEGF-A, endothelial cells, vascularization, induced pluripoten stem cells, nephrogenesis, angiogenesis

## Abstract

Stem cell–derived kidney organoids have been shown to self-organize from induced pluripotent stem cells into most important renal structures. However, the structures remain immature in culture and contain endothelial networks with low connectivity and limited organoid invasion. Furthermore, the nephrons lose their phenotype after approximately 25 days. To become applicable for future transplantation, further maturation *in vitro* is essential. Since kidneys *in vivo* develop in hypoxia, we studied the modulation of oxygen availability in culture. We hypothesized that introducing long-term culture at physiological hypoxia, rather than the normally applied non-physiological, hyperoxic 21% O_2_, could initiate angiogenesis, lead to enhanced growth factor expression and improve the endothelial patterning. We therefore cultured the kidney organoids at 7% O_2_ instead of 21% O_2_ for up to 25 days and evaluated nephrogenesis, growth factor expression such as VEGF-A and vascularization. Whole mount imaging revealed a homogenous morphology of the endothelial network with enhanced sprouting and interconnectivity when the kidney organoids were cultured in hypoxia. Three-dimensional vessel quantification confirmed that the hypoxic culture led to an increased average vessel length, likely due to the observed upregulation of VEGFA-189 and VEGFA-121, and downregulation of the antiangiogenic protein VEGF-A165b measured in hypoxia. This research indicates the importance of optimization of oxygen availability in organoid systems and the potential of hypoxic culture conditions in improving the vascularization of organoids.

## Graphical Abstract

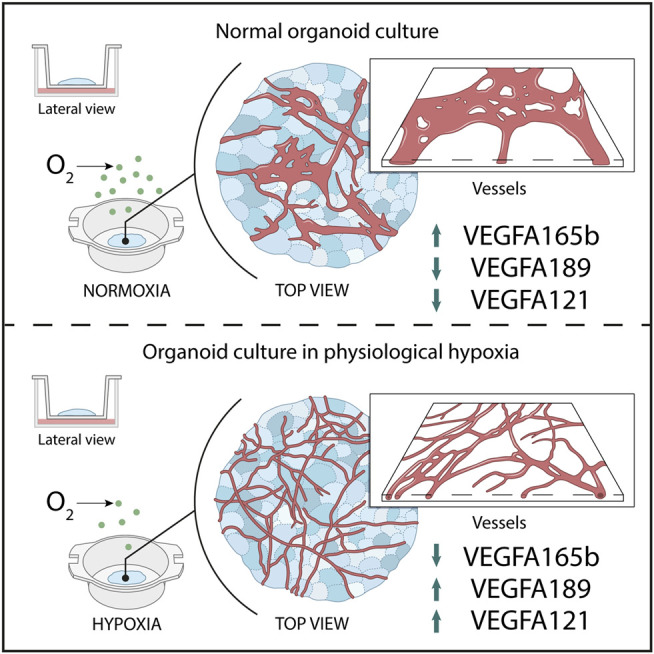

## Introduction

Organoid models have become an irreplaceable alternative to two-dimensional cell culture because of their greater cellular and architectural complexity, which is alike native tissue. Induced pluripotent stem cells can be differentiated into kidney organoids that develop nephrons resembling capillary loop–stage nephrons. (Takasato et al., 2016) The large variety of renal cell types they possess, similar to the developing kidney (Schumacher et al., 2021), makes them promising models for regenerative medicine, drug testing and developmental biology (Gupta et al., 2021). However, these organoids have several drawbacks—such as the limited culture duration, loss of nephrogenic potential, immaturity and lack of vasculature (Nishinakamura, 2019; Takasato and Wymeersch, 2020) —perhaps due the lack of an *in vivo*–like culture environment, which is increasingly being investigated. (Rossi et al., 2018) Moreover, kidney organoids are one of the largest organoid models, growing up to 1.5 mm in thickness. (Takasato et al., 2016) This draws attention to one aspect of a physiological environment, which is the oxygenation of the tissue.

Cell culture in hypoxia, defined as a state where cells no longer have sufficient oxygen available to use oxidative phosphorylation to generate ATP (Nishinakamura, 2019; Takasato and Wymeersch, 2020), has been performed for more than a decade, for example to maintain stem cells (Ma et al., 2009). Hypoxia is also known to act as a morphogen in cell communication of various cell lineages and for certain cell types to partially determine cellular differentiation. (Wörsdörfer and Ergün, 2021) If not optimized to the model system, hypoxia is known to have detrimental effects in cell and tissue culture. Nevertheless, to date, there is little knowledge on the effects of hypoxia in organoid cultures. Briefly, hypoxic culture (5% O_2_) of intestinal organoids was considered detrimental due to a reduced number of crypts. (Okkelman et al., 2017) In contrast, microwell-cultured kidney organoids cultured for 24 h in hypoxia (1 and 3% O_2_) showed enhanced functionality measured by secretion of erythropoietin (EPO). (Ding et al., 2020) These results argue that oxygen levels critically affect maturation of organoids, in a model-sensitive manner.

Kidney organoids are cultured, like explanted kidneys (Grobstein, 1956), at an air–liquid interface and therefore are directly exposed to incubator air (21% O_2_), commonly defined as normoxia. However, it has been previously well described that 21% O_2_ is non-physiological. (Place et al., 2017) For this reason, and the fact that the physiological environment of the developing kidney is hypoxic, we do consider 21% O_2_ to be hyperoxic and non-physiological. This is particularly the case for endothelial cells, which largely reside on the organoids’ surface. Hyperoxia is known to negatively impact kidney development *in vivo,* such as significantly reducing the size of the nephrogenic zone and glomeruli. (Buchholz et al., 2016) *In vitro*, the detrimental effects of hyperoxic cell and tissue culture are also well established (Li et al., 2004; Jagannathan et al., 2016), such as the formation of reactive oxygen species in endothelial cells (Brueckl et al., 2006). By contrast, there is significant evidence that hypoxia enhances the proliferation of endothelial cells as well as stem cell differentiation towards an endothelial lineage. (Han et al., 2010; Prado-Lopez et al., 2010; Salomon et al., 2013; Bekhite et al., 2014; Yuan et al., 2015; Shang et al., 2019; Podkalicka et al., 2020) Murine metanephric explants showed enhanced endothelial cell proliferation when cultured in hypoxia (3% O_2_). (Tufro-McReddie et al., 1997; Loughna et al., 1998) Furthermore, the mammalian uterus is hypoxic and fetal organs develop in hypoxic environments. (Fischer and Bavister, 1993; Lee et al., 2001) Therefore, modulating the oxygen concentrations could be a step towards *in vivo*–like kidney organoid culture with improved vascularization.

In developing kidneys in the hypoxic mammalian uterus (Fischer and Bavister, 1993; Fajersztajn and Veras, 2017), nephrogenesis starts in the avascular nephrogenic zone. (Hemker et al., 2016; Gerosa et al., 2017) Later in the capillary loop stage of nephrogenesis, mainly angiogenesis and to a lesser extent vascularization start to take place. (Nishinakamura, 2019) Only when blood vessels enter the kidney and new vessels are formed, oxygen levels increase to finally reach 4–9.5 kPa (30–71 mmHg) in the adult human cortex and 2 kPa (15 mmHg) in the adult human medulla. (Keeley and Mann, 2019) Due to the invasive nature of the measurements, the oxygen tensions in developing human cortex and medulla have not been determined. Recapitulating this *in vivo* hypoxic environment in the kidney organoid culture could enable the transcription of genes essential in kidney organogenesis. Below 5% O_2_, binding of prolyl hydroxylases to cytoplasmic hypoxia-inducible factor alpha (HIFα) is inhibited, leading to reduced or inhibited proteosomal degradation. Consequently, HIFα rapidly accumulates, translocates into the nucleus and its dimerization with HIFβ is initiated. (Hemker et al., 2016) Dimer binding to hypoxia responsive element (HRE) promoters leads to the transcription of a variety of genes. While this process occurs in all tissue types, it is known that various HIF-regulated genes are implicated in angiogenesis and vascularization in kidney organogenesis. In kidney development, nuclear HIF translocation occurs in metanephric mesenchyme and subsequently in podocytes, leading to VEGF-A transcription, which attracts endothelial cells to vascularize the nephrons. (Tufró, 2000; Bernhardt et al., 2006).

Developmentally, kidney organoids, normally cultured at non-physiological hyperoxic 21% O_2_ (≈160 mmHg), are comparable to kidneys in the first to early second trimester. (Takasato et al., 2015) Since, however, the oxygen concentrations in the developing human kidney are unknown, we hypothesized whether a culture at 7% O_2_ (≈53 mmHg), comparable to the adult human cortex, would lead to intra-organoid oxygen concentrations more closely resembling developing non-vascularized kidneys *in vivo,* and initiate angiogenesis in the organoids. After up to 25 days of culture, we analyzed nephrogenesis, the expression of angiogenic markers (particularly VEGF) and endothelialisation to determine whether hypoxic cultures of the organoids improved these characteristics compared to cultures at 21% O_2_. We found that long-term hypoxic culture enhances endothelial patterning and sprouting and consequently could enhance kidney organoid cultures towards a more *in vivo*–like model.

## Materials and Methods

### Induced Pluripotent Stem Cell Differentiation and Kidney Organoid Culture

**FIGURE 1:**
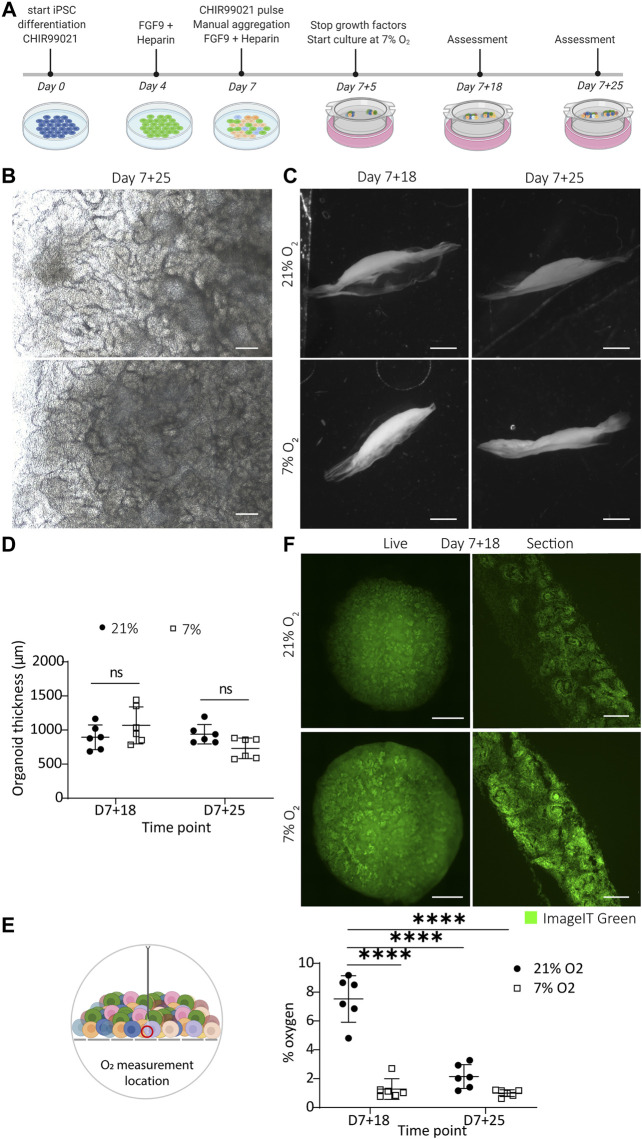
Kidney organoids cultured in uterine-like hypoxic environment structurally form like their normoxic counterparts without the appearance of a hypoxic core. **(A)**. Schematic representation of the iPSC differentiation and organoid culture. Human iPSCs are differentiated for 7 days, after which aggregates are spotted on the air–liquid interface and cultured as organoids for up to 25 additional days (termed day 7 + 25). **(B)**. Brightfield images display comparable morphologies of the organoids cultured in 7 and 21% O_2_ at the culture endpoint day 7 + 25. Scale bar: 200 µm. **(C)**. Darkfield images show that the macro-anatomy of the organoids is comparable in both oxygen concentrations. Scale bar: 2 mm. **(D)**. Quantification of the organoid thickness from the darkfield images shows no difference between 21% O_2_ and 7% O_2_. (*n* = 2, *N* = 3). **(E)**. Oxygen measurement in the bottom part of the organoids towards the transwell filter shows that organoids in 7% oxygen have a comparable oxygen concentration to their normoxic counterparts at day 7 + 25, which is significantly lower compared to the normoxic condition at day 7 + 18. (*n* = 2, *N* = 3; **** = *p* ≤ 0.0001) **(F)**. Organoids (left column) and cryosections of their central core (right column) stained on day 7 + 18 with the hypoxia dye ImageIT Green that produces a fluorescent signal below 5% O_2_. The organoids display no hypoxic core but the dye stains all nephrons. Scale bar “live”: 1 mm, scale bar “section”: 100 µm.

Induced pluripotent stem cells (iPSCs) were differentiated and the organoids were cultured according to the previously published protocol by van den Berg, Ritsma (van den Berg et al., 2018) (Figure 1A). Briefly, the iPSC line LUMC0072iCTRL01 (male fibroblasts reprogrammed using RNA Simplicon reprogramming kit, Millipore) obtained from the hiPSC core facility at the Leiden University Medical Center, were passaged biweekly and maintained in 3 ml of Essential 8 Medium (Thermo Fisher Scientific) supplemented with 1% penicillin–streptomycin in 6-well plates coated with truncated recombinant human vitronectin (Thermo Fisher Scientific). The iPSC line was previously assessed for pluripotency and normal karyotype. (Geuens et al., 2021) For differentiation, 80,000 cells per well were seeded into a six well plate and differentiated according to the published protocol in organoid culture medium composed of STEMdiff APEL2 medium (STEMCELL Technologies), supplemented with 1% antibiotic-antimycotic and 1% protein-free hybridoma medium II (PFHM 2) (Thermo Fisher Scientific) and the respective growth factors and small molecules (8 µM GSK-3 inhibitor CHIR99021 (R&D Systems), 200 ng/ml FGF9 (fibroblast growth factor 9; R&D Systems), and 1 μg/ml heparin (Sigma-Aldrich)). After 7 days of differentiation, the cells were aggregated by centrifugation and spotted on transwell tissue culture plates with 0.4 μm pore polyester membrane inserts (Corning) and cultured at an air–liquid interface. For an additional 5 days, denoted as day 7 + 5, the organoids were cultured in organoid medium containing the growth factors and small molecules. From day 7 + 5 onwards, the organoids were cultured in organoid culture medium either in a hypoxia incubator (37°C, 5% CO_2_, 7% O_2_) or in normoxia (37°C, 5% CO_2_, 21% O_2_). The medium was refreshed every 2 days. At day 7 + 18 and day 7 + 25, the organoids were assessed.

### Darkfield Imaging

The organoids were fixed at day 7 + 18 and day 7 + 25 in 2% (v/v) paraformaldehyde (20 min, 4°C) and were subsequently embedded in 10% gelatin in PBS (phosphate-buffered saline) in a cryomold. Once hardened, the gel was pealed out of the mold and placed on a darkfield imaging stage with circular illumination (Nikon). Images were acquired using a 1 × air objective on a Nikon SMX25 automated stereomicroscope with a customized Nikon darkfield illumination holder.

### Cryopreservation and Cryosectioning

After fixing the organoids at day 7 + 18 or day 7 + 25 in 2% (v/v) paraformaldehyde (20 min, 4°C), the organoids were cryoprotected. The organoids were immersed in 15% (w/v) sucrose in 0.1 M phosphate buffer containing 0.1 M dibasic sodium phosphate and 0.01 M monobasic sodium phosphate in MilliQ water (24 h, 4°C, rotating), and subsequently in 30% (w/v) sucrose in 0.1 M phosphate buffer (48 h, 4°C, rotating). After cryoprotection, the organoids were embedded in freezing medium (15% (w/v) sucrose and 7.5% (w/v) gelatin in 1 M phosphate buffer) and hardened on ice. Freezing was performed in an isopentane bath in liquid nitrogen and the blocks were stored at −30°C until cryosectioning at −18°C into 12 µm–thick sections. The sections were stored at −80°C until use.

### Oxygen Measurement

The oxygen concentration within the organoids was measured using an optical oxygen microsensor (PM-PSt7, Presens in Regensburg, Germany). The probe was calibrated according to the manufacturer’s instructions. Briefly, the pressure settings were set to the elevation of the lab (997 kPa) and the probe was calibrated in an oxygen-depleted solution (water containing 70 mM sodium sulfite and 500 mM cobalt nitrate) followed by an oxygen-enriched solution (water connected to a room air valve). All solutions were equilibrated to room temperature before calibration and room temperature (20°C) was put as standard in the software. At day 7 + 18 and day 7 + 25, organoids in both normoxia and hypoxia were measured using the calibrated sensor. The sensor was fixed to a micromanipulator and was inserted into the bottom of the organoid, approximately 1 mm in depth, close to the insert membrane of the transwell. The incubator was kept closed to ensure a stable gas concentration and measurements were continued until the signal reached a steady state (taking approximately 30–90 min).

### Hypoxia Imaging

Hypoxia was measured in living organoids using the Image-iT Green Hypoxia Reagent (Thermo Fisher Scientific), which produces a green fluorescent signal below 5% O_2_. The organoid was fully immersed in 5 µM ImageIT Green reagent dissolved in organoid culture medium for 4 h in normoxia. Subsequently, the staining solution was replaced with fresh medium and the organoids were moved to the incubator set to either the 7% O_2_ or the 21% O_2_ for 6 h, after which they were fixed for cryosectioning or imaged live. For live imaging, the organoids were imaged using a 10× air objective on an automated Nikon Eclipse Ti-E equipped with a spinning disk with a 70 µm pore size, a Lumencor Spectra X light source and Photometrics Prime 95B sCMOS camera. For cryosectioning, the method in Section 2.3 was applied. The sections were imaged with the same microscope using a 20× air objective and a 40× oil objective. All images were processed using Fiji (Schindelin et al., 2012), in which the rolling ball function was applied in the case of poor signal to noise ratio.

### Immunofluorescence

The cryosections were warmed to RT (room temperature) and subsequently incubated in pre-warmed PBS (15 min, 37°C), to remove the sucrose and gelatin. Next, the cryosections were blocked with PBS containing 0.2% (v/v) Tween, 10% (w/v) BSA (bovine serum albumin) and 0.1 M glycine (20 min, RT) and incubated in primary antibodies (Supplementary Table S1) diluted in the dilution buffer of PBS containing 0.2% (v/v) Tween, 1% (w/v) BSA and 0.1 M glycine (overnight, 4°C). After washing in PBS containing 0.2% (v/v) Tween, the slides were incubated with appropriate secondary antibodies (Supplementary Table S1) diluted in the dilution buffer (1 h, RT). Finally, the slides were washed and mounted with Prolong Gold (Thermo Fisher Scientific). After curing for 2 days, imaging was performed on the automated Nikon Eclipse Ti-E microscope using a 20× air and a 40× oil objective. All images were processed using Fiji (Schindelin et al., 2012), in which the rolling ball function was applied in the case of poor signal to noise ratio.

### Luminex Assay

The VEGF-A concentration in the culture medium was analyzed using a VEGF-A Human ProcartaPlex Simplex Kit (Cat. no. EPX01A-10277-901, Invitrogen), specific for the detection of VEGF-A165. The medium was collected on days 7 + 7, 7 + 12, 7 + 17, 7 + 21, and 7 + 24 and centrifuged (10 min, 4°C, 239 x g). The supernatant was immediately stored at −80°C. The assay was performed according to the manufacturer’s instructions. In brief, samples were diluted 1:50 in universal assay buffer and 50 µL was added to the wells containing the antibody-coupled beads, along with the standards provided with the assay (30 min, RT, shaking). After an overnight incubation (4°C) and a final incubation (30 min, RT, shaking), detection antibody–biotin reporters were added to each well (30 min, RT, shaking). Next, fluorescent conjugate streptavidin–phycoerythrin was added (30 min, RT, shaking). After a final washing step, the beads were resuspended in 120 μL reading buffer. Fluorescence intensities were measured using a Luminex100 instrument (Bio-Rad) which was calibrated before each use. Data acquisition was done with the Bio-Plex Manager 6.0 software. The data were normalized to the standard curve dilutions delivered with the kit according to manufacturer’s instructions.

### Western Blot

After snap freezing in liquid nitrogen, the organoids were resuspended in 70 µL RIPA (radioimmunoprecipitation assay) lysis buffer (Sigma-Aldrich) supplemented with phosphatase inhibitor tablets PhosSTOP (Sigma-Aldrich) and protease inhibitor cOmplete ULTRA Tablets (Sigma-Aldrich). The protein concentrations were determined using a Pierce BCA Protein Assay Kit (Thermo Fisher Scientific) according to the manufacturer’s protocol. Per well, 15 µg of protein was loaded. The migration was performed in migration buffer (Tris [tris(hydroxymethyl)aminomethane]-EDTA (ethylenediaminetetraacetic acid), SDS (sodium dodecyl sulfate), glycine, Bio-Rad) at 120 V. Subsequently, the proteins were transferred onto a nitrocellulose membrane at 350 mA for 90 min in a transfer buffer (Tris-base, glycine, SDS, 20% methanol). After the transfer, the membranes were blocked in blocking buffer [TBS (Tris-buffered saline), 0.1% Tween (v/v), 5% BSA (w/v)] (1 h, RT, shaking). Next, the membranes were incubated with primary antibodies (Table 1) (overnight, 4°C, shaking) in TBS-Tween, 5% BSA (w/v). The membranes were washed in TBS-Tween and incubated with the peroxidase-conjugated secondary antibody (Bio-Rad, Table 1) for 1 h at RT. The membranes were incubated with Clarity Western ECL substrate (Bio-Rad) and were developed using a ChemiDoc (Bio-Rad). The protein bands were quantified by densitometry using ImageJ, normalized to GAPDH. (Schneider et al., 2012).

### RNA Isolation and qPCR

Organoids stored in TRIzol at −80°C were thawed and pipetted vigorously to homogenize the samples. Then, 500 µL was transferred to a Phasemaker tube (Thermo Fisher Scientific) after which, 100 µL of chloroform were added, shaken thoroughly and incubated for 5 min at RT. The mixture was centrifuged at 12,000 × g (15 min, 4°C). The aqueous phase was carefully transferred to a new microcentrifuge tube containing 250 µL isopropanol and 1 µL glycogen. After an additional centrifugation step (15 min, 4°C, 12,000 × g) the pellet was washed twice with 200 mM NaOAc in 75% ethanol. The supernatant was discarded, and the pellet was dried at 55°C, resuspended in 25 µL nuclease-free water. CDNA was synthesized using the iScript cDNA synthesis kit (Bio-Rad) and 500 ng/μL of RNA was loaded per sample. Quantitative PCR was carried out with iQ SYBR Green Supermix (Bio-Rad), 5 ng cDNA per reaction, on a CFX96TM Real-Time system (Bio-Rad). Primer sequences (Supplementary Table S2) were verified using total human kidney RNA (Takara Bio). *PSMB4* was determined as the most stable housekeeping gene using the method described by Xie, Xiao (Xie et al., 2012), and *GAPDH* was used as a second housekeeping gene to ensure valid results. The data were normalized to *PSMB4* and plotted relative to the expression of the control samples day 7+18 normoxia. Each data point represents one organoid of three distinct iPSC differentiations. The statistics were performed on the log (fold change) and plotted onto the fold change graphs.

### Whole Mount Immunofluorescence, Tissue Clearing and Automated Imaging

Whole organoids were fixed in 2% paraformaldehyde (20 min, 4°C) and blocked in PBS containing 10% goat serum, 0.1 M glycine and 0.5% Triton X-100 (overnight, 4°C, shaking). They were incubated in primary antibodies (Supplementary Table S1) diluted in PBS containing 10% goat serum, 0.1 M glycine and 0.06% Triton X-100 (3 days, 4°C, shaking). After two washes in 0.3% Triton X-100 in PBS (2 h, RT, shaking), the organoids were incubated with the appropriate secondary antibodies (Supplementary Table S1) diluted in PBS containing 1% goat serum, 0.1 M glycine and 0.06% Triton X-100 (3 days, 4°C, shaking). After two washes (2 h, RT, shaking), the organoids were cleared using the method of Klingberg, Hasenberg (Klingberg et al., 2017). Briefly, the organoids were dehydrated in a series of 30–100% molecular biology–grade ethanol and submerged in ethyl cinnamate (overnight, RT followed by 1 h, 37°C). Imaging was done immediately after on an automated Nikon Eclipse Ti-E with the 70 µm spinning disk in place. Imaging was automated using a Nikon JOBS tool. Briefly, a 10× air objective was used for automated detection of the organoid, while a 20× air objective with an extra-long working distance was used to image at higher resolution with a z increment size of 5 µm.

### Automated Image Processing and Vessel Quantification

Immunofluorescence of VEGF-A on cryo-sections was quantified using FIJI. The FIJI default threshold was applied on the DAPI channel and the Renyi Entropy threshold on the VEGF-A channel. Next, the area of both thresholded signals was measured and the area of VEGF-A was normalized to the area of DAPI signal to correct for different sizes of sections. The results are presented as percent area.

Whole mount images were processed within a processing and quantification JOB specifically made for these samples, integrated in the NIS-Elements AR (Advance Research) (Nikon) software. In summary, background was removed using the clarify AI tool prior to the segmentation of the CD31 signal. The CD31 signal was thresholded by intensity and segmented by measuring the longest medial axis in 3D. Falsely segmented pixels, due to intense background signal, were excluded by circularity and minimal size if needed. The volume of the segmented signal was measured in 3D for the full organoid. The volume of the total organoid was measured by segmenting the organoid based on the strong background. The percentage of vascularization was determined by calculating the CD31+ cell volume as a fraction of the total organoid volume. Furthermore, the segmented CD31 signal was skeletonized to measure the cumulative and average length of all fragments in 3D. Stepwise details on the used JOB can be found in Supplementary Figure S1.

### Statistical Analysis

All statistical analyses were performed using GraphPad Prism 9. For all immunofluorescence protein analyses, three organoids (*n* = 3) each arising from one of three independent organoid cultures (*N* = 3) were assessed. One organoid (*n* = 1) arising from one of three (*N* = 3) independent organoid cultures was quantified in 3D. For the gene expression analysis and Luminex assay, three organoids (*n* = 3) each arising from one of two independent organoid cultures (*N* = 3) were analyzed. Two organoids (*n* = 2), each arising from one of three independent organoid cultures (*N* = 3) were assessed for the darkfield measurement and oxygen measurement. Individual samples were excluded only for technical failures. For each figure, the exact N and n are reported in the figure caption. A two-way ANOVA was performed for each experiment to assess the contribution of both row (time point) and column (oxygen concentration) factors. All *p*-values can be found in the Results section. Statistical significance was only concluded for *p*-values below 0.05.

## Results

### Macro-Morphology and Oxygen Concentrations in Normoxic and Hypoxic Organoid Culture

To replicate the *in vivo* hypoxic environment, we cultured kidney organoids in 7% O_2_ and compared them to their counterparts cultured in 21% O_2_ (Graphic Abstract). Brightfield analysis revealed no differences in macro morphology, density or size until day 7 + 25 (Graphic Abstract, Supplementary Figure S2). Darkfield imaging was performed to assess the shape and thickness of the organoids (Graphic Abstract). No statistically significant differences in thickness were found comparing organoids cultured in normoxia and hypoxia (*p* = 0.839; Graphic Abstract).

To quantify the oxygen concentration, we inserted an optical microsensor into the bottom of the organoid (close to the filter). In normoxia, the lowest oxygen concentration we measured was 7.53 ± 1.47% on day 7 + 18, which significantly decreased to 2.15 ± 0.66% by day 7 + 25 (*p* < 0.0001). This value at day 7 + 25 was not significantly different from the oxygen concentration measured in the organoids cultured in hypoxia on day 7 + 18 (1.28 ± 0.76%; *p* = 0.439) or day 7 + 25 (1.00 ± 0.21%; *p =* 0.211) (Graphic Abstract).

To investigate whether the organoids had a hypoxic core, we imaged living organoids and cryosections at the center of the organoids with a hypoxia dye (ImageIT Green) (Graphic Abstract). The cryosections, cut through the center of the organoids, revealed a higher intensity (meaning lower O_2_) in hypoxia. We saw the dye localized largely to nephron structures and less to the surrounding stroma. We did not see evidence of a hypoxic core in the center of any organoids.

**FIGURE 2:**
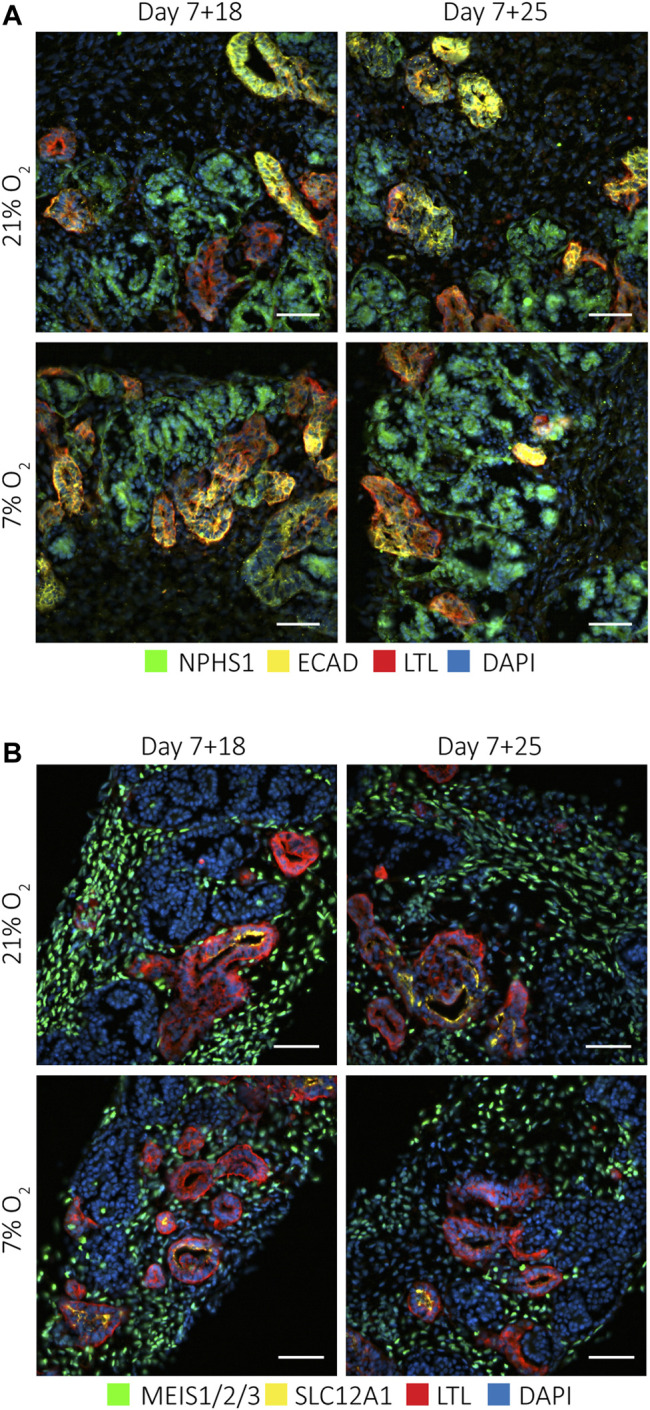
Kidney organoids cultured at 7 and 21% O_2_ develop the same cell types. Immunofluorescence staining for **(A)**. podocytes (NPHS1; green), proximal tubules (LTL; red) and distal tubules (ECAD; yellow) and nuclei (DAPI; blue), and **(B)**. interstitial cells (MEIS1/2/3; green), loop of Henle (SLC12A1; yellow) and proximal tubules (LTL; red), showed these different cell types in all conditions. (*n* = 3, *N* = 3). Scale bars: 50 µm.

Next, we explored if culture in hypoxia would affect the differentiation and organization of various cell types in the kidney organoids. To assess this, we stained vertically cut cryosections throughout the whole organoids for well-established markers to label the major cell types found in kidney organoids. We found no differences in either the presence of the cell types or their organization into proximal tubules (LTL), distal tubules (ECAD), loop of Henle (SLC12A1) or glomeruli (NPHS1-positive podocytes) (Figures 2A,B).

### Nephrons Express Varied Levels of Nuclear HIF1α and a Constant Nuclear Expression of HIF2α

**FIGURE 3:**
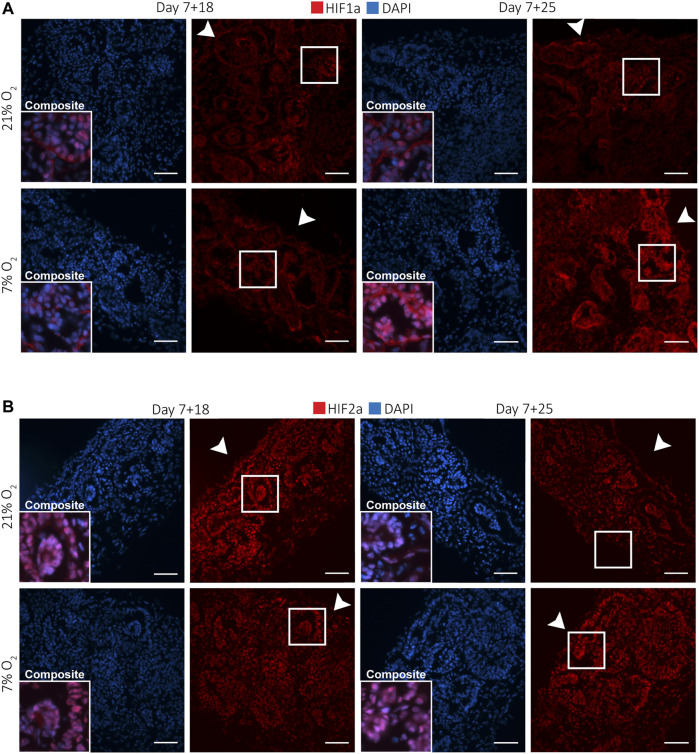
Kidney organoids cultured in both 7 and 21% O_2_ show differential nuclear translocation of HIF1a and HIF2a. **(A)**. Immunofluorescence staining for HIF1a (red) and DAPI (blue) shows increased nuclear translocation in podocytes (composite), particularly in 7% O_2_ at day 7 + 25. Scale bar: 50 µm. **(B)**. HIF2a (red) and DAPI (blue) immunofluorescence staining shows nuclear translocation in most cells within the organoid irrespective of the oxygen concentration or time point. (*n* = 3, *N* = 3). Scale bar: 50 µm. Arrowheads indicate the (air-exposed) surface of the organoids.

Nuclear HIF translocation during kidney development is crucial for nephrogenesis and angiogenesis. We investigated which cells showed responsiveness to hypoxia in terms of nuclear translocation of HIF1α and HIF2α. For this, we performed immunofluorescence staining of HIF1α and HIF2α on vertically cut cryosections throughout the whole kidney organoids at days 7 + 18 and 7 + 25 (Figure 3). Qualitative analysis showed that HIF1α was differentially expressed in hypoxia and normoxia (Figure 3A). At day 7 + 25, particularly the podocytes had more nuclear translocation in both normoxia and hypoxia compared to other cell types. Furthermore, in normoxia, podocytes at the bottom of the organoid, towards the medium interface, expressed nuclear HIF1α, while in hypoxia this was seen in podocytes throughout the whole organoid. In all conditions and time points, interstitial cells and most tubules did not express nuclear HIF1α. HIF2α was expressed in the nuclei of all cells of the kidney organoids at days 7 + 18 and 7 + 25 (Figure 3B). Particularly the interstitial cells had heterogeneous expression. We did not detect differences between organoids cultured in normoxia or hypoxia in terms of HIF2α nuclear translocation.

### VEGF-A Protein Expression in Proximal Tubules

**FIGURE 4:**
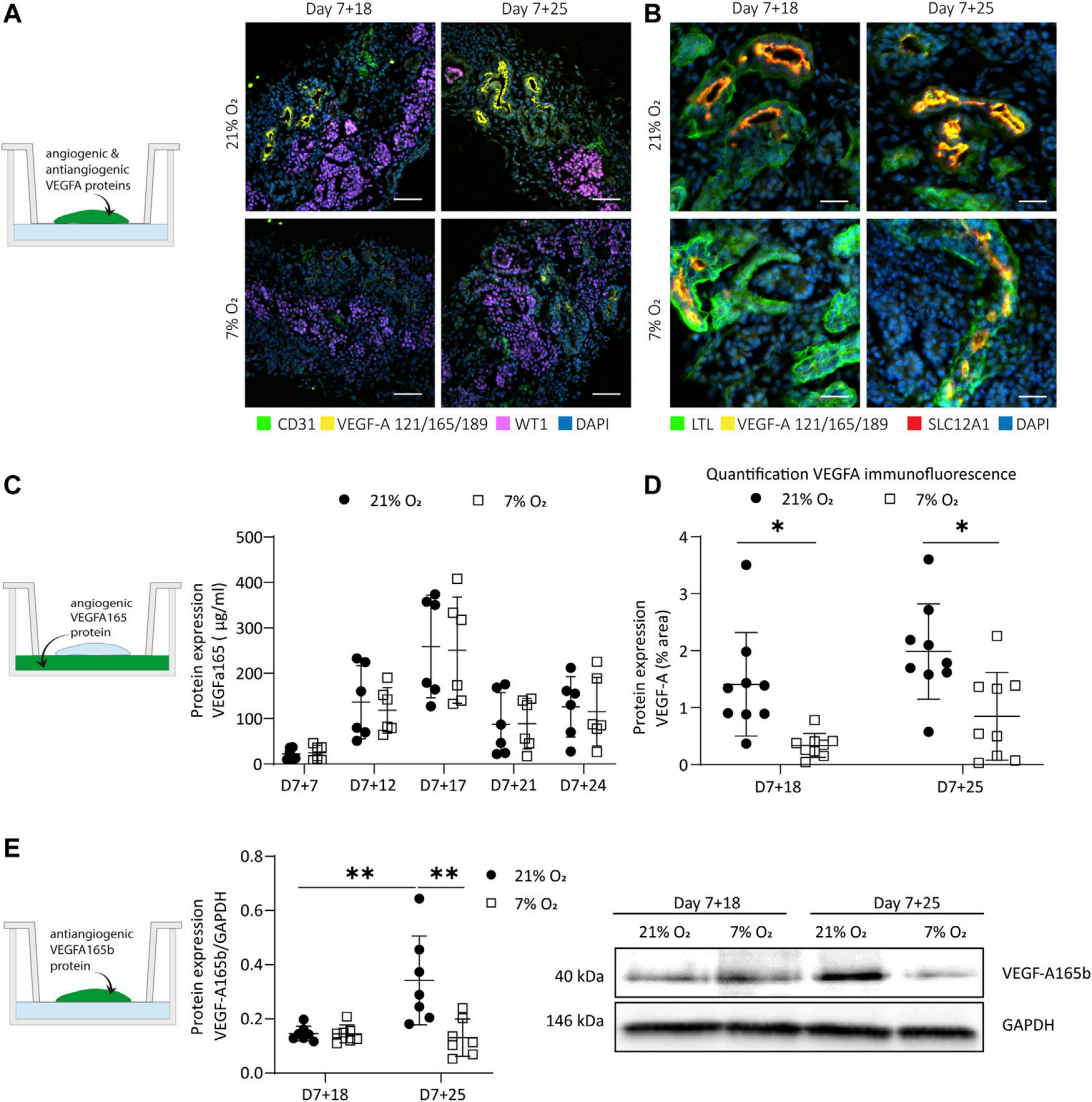
Expression of hypoxia-responsive VEGF-A in organoids cultured in 21 and 7% O_2_. **(A)**. Immunofluorescence for VEGF-A (121, 165, 189 isoforms; yellow), endothelial cells (CD31; green), podocytes (WT1; magenta) and nuclei (DAPI; blue) displays a reduction of VEGF-A in 7% O_2_ compared to 21% O_2_ at both day 7 + 18 and day 7 + 25. (*n* = 3, *N* = 3). Scale bar: 50 µm. **(B)**. Immunofluorescence shows localization of VEGF-A to the apical side of proximal tubules co-positive for loop of Henle marker (SLC12A1; red). (*n* = 3, *N* = 3). Scale bar: 25 µm. **(C)**. VEGF-A165 protein expression measured in the culture medium was differentially expressed over time, but not significantly different in hypoxia compared to normoxia. (*n* = 3, *N* = 2). **(D)**. Quantification of immunofluorescence images confirm that VEGF-A was significantly lower expressed in hypoxia compared to normoxia. (*n* = 3, *N* = 3) **(E)**. The anti-angiogenic VEGF-A165b isoform was significantly upregulated over time in normoxia and significantly downregulated in hypoxia compared to normoxia at day 7 + 25. (*n* = 2, *N* = 3). *= *p* ≤ 0.02, ** = *p* < 0.003.

VEGF-A is a major angiogenic factor primarily produced in distal tubules, collecting duct and podocytes. (Freeburg et al., 2003) It is regulated by hypoxia by the binding of nuclear, dimerized HIFs to the HRE promotor. We performed immunofluorescence on vertically cut cryosections throughout whole organoids to determine if there was a difference in expression between normoxia- and hypoxia-cultured organoids (Figure 4A), and which cell types expressed VEGF-A (Figure 4B). We found expression on the apical side of some tubular structures, mainly in normoxia and to a lesser extent in hypoxia (Figures 4A,B). VEGF-A was significantly less expressed in hypoxia compared to normoxia at both time points (*p* = 0.02 at d7+18, *p* = 0.013 at d7+25), but there was no difference between the two time points (*p* = 0.362 in normoxia, *p* = 0.461 in hypoxia) (Figure 4D). The VEGF-A+ tubules in both normoxia and hypoxia co-expressed both SLC12A1 marking the loop of Henle (thick ascending limb) and LTL marking proximal tubules (Figure 4B). We validated this co-expression of SLC12A1, LTL and VEGF-A on adult kidney sections and in scRNA sequencing datasets of organoids (Supplementary Figure S3). Glomerular expression of VEGF-A in the normoxia and hypoxia organoids rarely found. We hypothesized that the lower VEGF-A expression in the proximal tubules in hypoxia could be due to a switch to a more soluble VEGF-A isoform or that the time point of secretion would be different. Therefore, soluble VEGF-A was measured using a Luminex assay validated for the VEGF-A165 isoform in the culture medium on days 7 + 7, 7 + 12, 7 + 17, 7 + 21, 7 + 24 (Figure 4C). There was an increase over time from day 7 + 7 until 7 + 17 for organoids in both hypoxia and normoxia. After day 7 + 17, the soluble VEGF-A decreased until day 7 + 21. There was no significant difference between organoids in normoxia or hypoxia (*p* = 0.544). The protein expression of the antiangiogenic isoform VEGF-A165b normalized to GAPDH expression was determined by Western blot (Figure 4E, Supplementary Figure S4). VEGF-A165b was significantly upregulated in normoxia from day 7 + 18 to day 7 + 25 (*p* = 0.003), while there was a significant reduction in hypoxia compared to normoxia at day 7 + 25 (*p* = 0.001).

### Differential Expression of Angiogenesis-Regulating Genes in Hypoxic Culture

**FIGURE 5:**
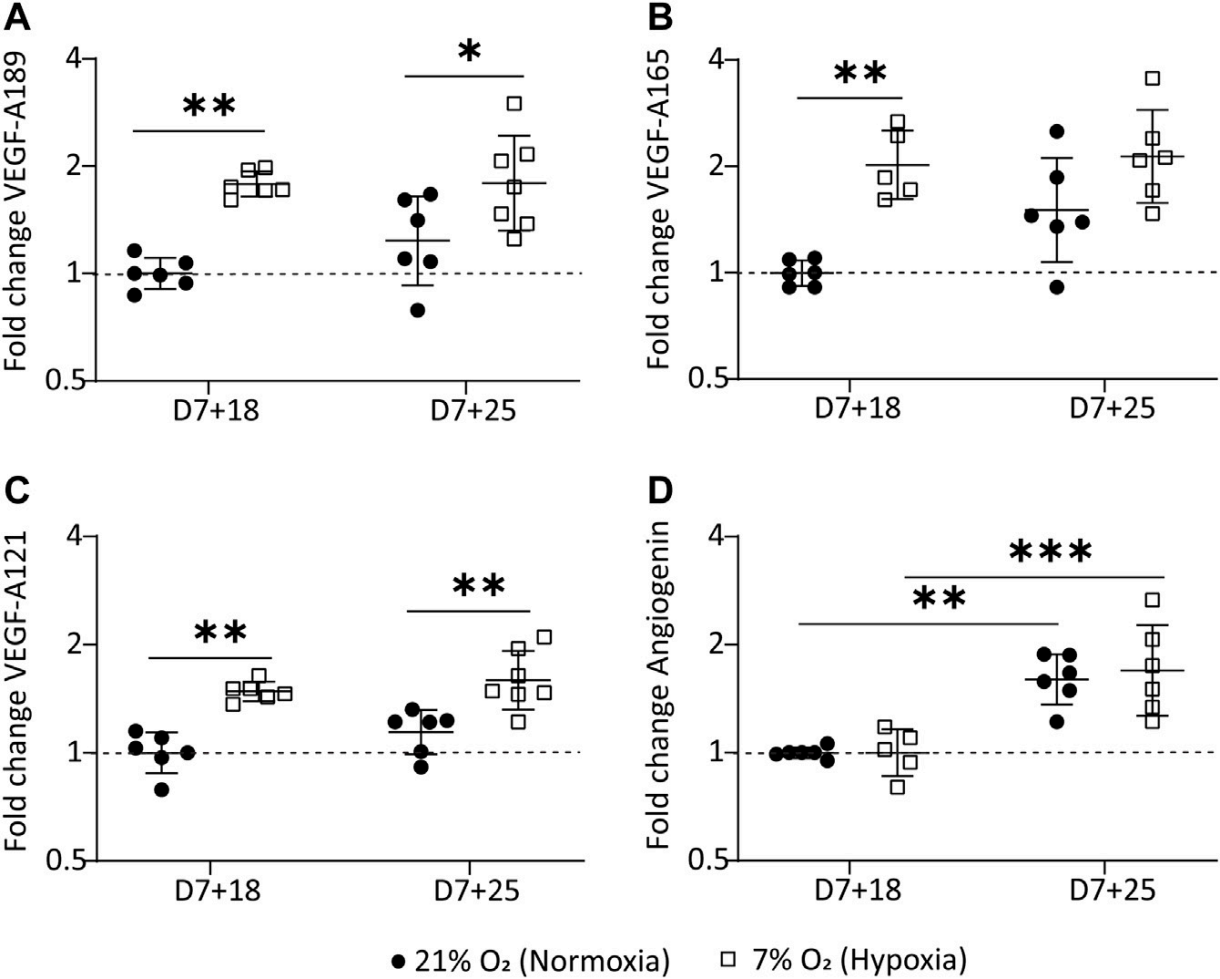
Hypoxic organoid culture differentially regulates VEGF-A variants. **(A)**. VEGF-189 is significantly upregulated at both time points in the hypoxic culture. **(B)**. VEGF-A165 is significantly upregulated at day 7+18 in the hypoxic culture and **(C)**. VEGF-121 is significantly upregulated at both time points in the hypoxic culture. **(D)**. Angiogenin mRNA is significantly upregulated over time in both the normoxic and hypoxic culture. The data is plotted relative to day 7+18 normoxia (n=3, N=3 with excluded technical failures). *= *p* ≤ 0.03, ** = *p* ≤ 0.002, *** = *p* ≤ 0.0004.

*VEGF-A* is known to exist in various splice variants with distinct biological functions. *VEGF-A165* is the most prominent variant, followed by *189* and *121* in the adult human kidney. (Vempati et al., 2014) We investigated *VEGF-A* variant expression in the organoids by quantitative polymerase chain reaction (qPCR). VEGF-A189 was significantly higher in hypoxia cultures than in normoxia, with a 1.8 fold-change (FC) at day 7+18 (*p* = 0.001) and 1.9 fold-change at day 7+25 (*p* = 0.032) relative to the control samples expression (day 7+18 normoxia) (Figure 5A). VEGF-A165 was significantly upregulated at day 7+18 relative to the control samples (FC = 2.06; *p* = 0.0014), but there was no significant difference between organoids in normoxia or hypoxia at day 7+25 (Figure 5B). VEGF-A121 was significantly upregulated in hypoxia compared to normoxia at both day 7+18 (FC: 1.49; *p* = 0.0004) and day 7+25 (FC: 1.617; *p* = 0.002) (Figure 5C). The ANOVA main effect analysis showed that time did have a limited effect on the observed differences in VEGF-A variant expression (VEGF-A189: *p* = 0.243; VEGF-A121: *p* = 0.085; VEGF-A165: *p* = 0.047) and could largely be attributed to the hypoxic culture (VEGF-A189: *p* < 0.0001; VEGF-A121: *p* < 0.0001; VEGF-A165: *p* = 0.0001).

We also determined mRNA expression of *ANG*, the gene encoding angiogenin, another hypoxia-regulated angiogenic factor, in our kidney organoid culture. *ANG* mRNA was significantly upregulated over time (*p* < 0.0001) but with no differences between hypoxia and normoxia cultures (Figure 5D).

### Hypoxia-Induced Angiogenesis: Homogenous Micro-vessel Formation and Sprouting

**FIGURE 6:**
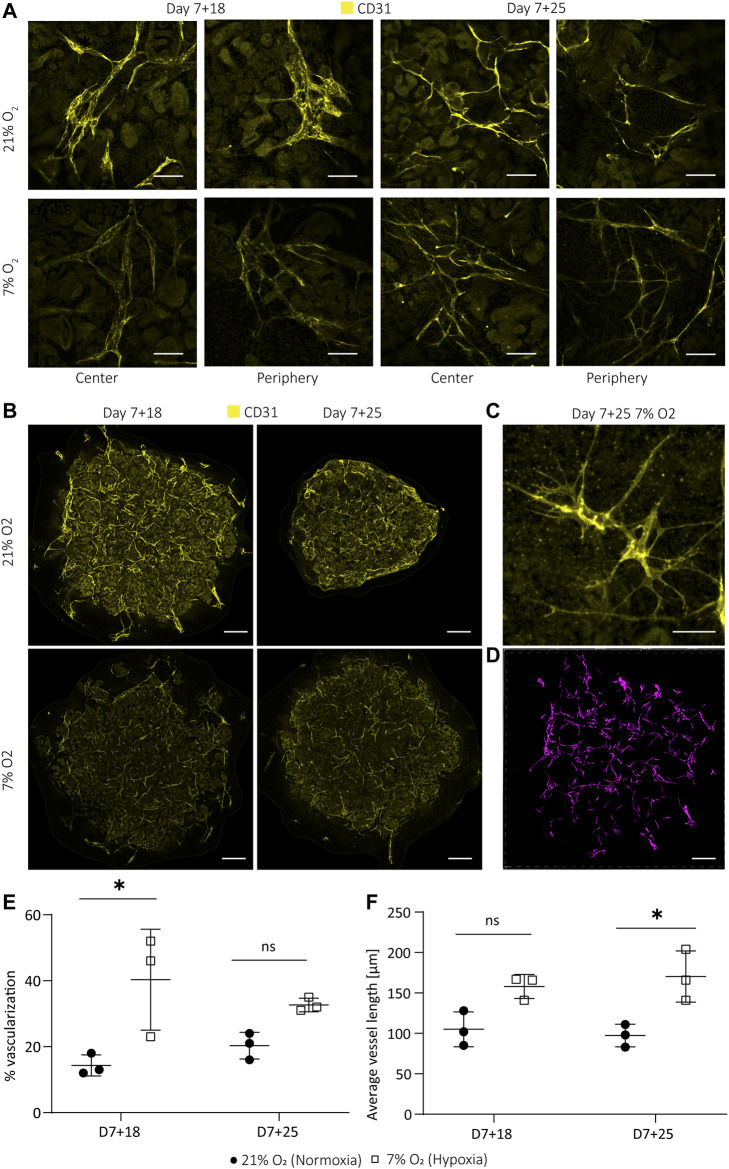
Hypoxic organoid culture promotes interconnected microvasculature formation and sprouting. **(A)**. The endothelial network (CD31; yellow) has a more homogeneous morphology and enhanced interconnectivity and branching in organoids cultured in 7% O_2_. **(B)**. Z-intensity projected images of whole mount imaged organoids show less intense CD31 staining, likely due to smaller vessel size. **(C)**. Hypoxia-induced sprouting of endothelial vessels at day 7 + 25. **(D)**. Example of 3D segmentation of endothelial network of a day 7 + 18 normoxia organoid. Scale bars: 50 µm **(A,C)**; 600 µm **(B,D)**. Quantification in 3D reveals **(E)**. an increase in the volume fraction of endothelial cells at day 7 + 18 in hypoxia and **(F)**. an increased average vessel length in hypoxia compared to normoxia at D7+25. (*n* = 1, *N* = 3). * = *p* ≤ 0.019.

Hypoxia is well known to enhance angiogenesis in both development and pathologies. (Otrock et al., 2007) In both, endothelial cells undergo sprouting to form new blood vessels. In the normoxia culture, endothelial cells largely reside on the surface of the organoids and are therefore directly exposed to the non-physiological oxygen concentrations (Supplementary Figure S5). We investigated the effect of hypoxia on the endothelial patterning by whole mount immunostaining with the endothelial marker CD31. Whole mount imaging and quantification in 3D revealed enhanced micro-vessel formation with more homogenous morphology, enhanced branching and sprouting in organoids cultured in hypoxia compared to normoxia (Figures 6A,C). We observed a reduced intensity of CD31 (not quantified) in hypoxia and normoxia at day 7 + 25 (Figure 6B). However, the endothelial phenotype was maintained, as confirmed by co-expression of CD31 and VE-cadherin in both normoxia- and hypoxia-cultured organoids at day 7 + 25 (Supplementary Figure S6). We set up an automatic 3D segmentation and quantification pipeline and found that hypoxia induced a significant increase in the fraction of endothelial cells of the total organoid volume (40.3 ± 12.50%) compared to normoxia (14.3 ± 2.60%) (*p* = 0.019; Figure 6E) at day 7 + 18 that was not observed at day 7 + 25 (*p* = 0.318). Interestingly, the average vessel length was significantly higher in hypoxia compared to normoxia at day 7 + 25 (hypoxia: 170 ± 26 μm; normoxia: 97 ± 11 μm; *p* = 0.014) (Figure 6D). There was no significant difference at D7+18 (*p* = 0.068). Three-dimensional segmentation additionally revealed that the endothelial network in organoids cultured in normoxia largely resided in a two-dimensional plane (parallel to the transwell), while in hypoxia culture, the network was mainly interconnected in three dimensions (Supplementary video 1 and 2).

## Discussion

Our aim was to investigate if a lower oxygen concentration applied to the kidney organoid culture that mimics the *in vivo* hypoxic environment during nephrogenesis could improve angiogenesis and organoid vascularization. The mRNA expression of endothelial markers (*CD31*, *KDR*) in the normoxic culture was downregulated over time (Supplementary Figure S7), indicating the need for stimuli to activate angiogenesis. We opted for a long-term hypoxic culture (20 days), because long-term hypoxia (hours to days, depending on the model) enhances the expression of angiogenic cytokines and growth factors such as PDGF and VEGF in comparison to acute hypoxia that is known to activate the release of inflammatory factors. (Michiels et al., 2000; Haddad and Harb, 2005) VEGF-A in particular is known to be crucial for endothelial cell differentiation, proliferation and migration (Abhinand et al., 2016), as well as glomerular capillary formation, podocyte survival and slit diaphragm integrity in autocrine podocyte signaling. (Guan et al., 2006) We therefore included VEGF-A mRNA and protein expression in our investigations.

Kidney organoids developed similarly in normoxic and hypoxic conditions. The hypoxic culture did not impair nephron specification and organization, nor organoid size (Figures 1B,C, Figures 2A,B), indicating that hypoxia did not interfere with normal organoid development. Our measurements of oxygen concentration on the bottom of the organoid culture plate (1.0 ± 0.21% in hypoxia on day 7 + 25) were lower compared to the poorly vascularized medullas in adults (1.9% O_2_ at atmospheric pressure derived from the measured 15 mmHg). (Keeley and Mann, 2019) We therefore expected our hypoxic culture to more closely resemble avascular, hypoxic kidneys during early development, although *in vivo* data are still lacking. (Freeburg and Abrahamson, 2003) This similarity indicates that growing organoids at 7% O_2_ could mimic the *in vivo* hypoxic environment and help study organoid maturation in a more physiological model.

In addition to oxygen levels, we found a similar transcriptional program activated in the kidney organoids as in the developing human kidney. Specifically, severe hypoxia and consequently HIF stabilization was found to be essential in nephrogenesis (41), with HIF-1α expressed in cortical and medullary collecting duct, nephrogenic zone and glomerular cells. (Bernhardt et al., 2006) While the collecting duct lineage does not exist in the organoids, podocytes deeper within the organoid, showed nuclear HIF-1α expression at day 7 + 18 in normoxia (Figure 3A). According to our measurements, these podocytes would reside in regions close to 7.53 ± 1.47% O_2_. Likely, only podocytes experiencing less than 5% O_2_ showed nuclear translocation of HIF-1α. (Hemker et al., 2016) Indeed, even peripheral podocytes of the hypoxia-cultured organoids, where the oxygen concentration was 1.0 ± 0.21% at the bottom of the organoids, showed nuclear HIF-1α translocation (Figure 3A). While certainly not all podocytes responded by nuclear HIF-1α translocation, the hypoxic culture clearly induces nuclear HIF-1α in podocytes throughout the organoids.

Comparable to HIF-1α, nuclear HIF-2α is important to *in vivo* kidney development, where it is known to be expressed only in interstitial cells and podocytes (13), as shown in week 14 fetal kidneys (33), as well as in developing tubules in newborns (41). In our kidney organoids, HIF-2α expression was not limited to interstitial cells and podocytes, but was expressed in all cell types examined in both hypoxia and normoxia (Figure 3B). There were clear differences in intensity of nuclear HIF-2α in interstitial cells within the organoids in normoxia and hypoxia, while the nephrons equally expressed nuclear HIF-2α. The reason for this observed difference is unclear. Clarification is also needed for why all interstitial cells, even in the most oxygenated condition (day 7 + 18 normoxia), do express nuclear HIF-2α. There is a need for further investigation, since recent findings in mice suggest that chronic HIF-2α expression in stromal progenitors impairs kidney development, in particular nephron formation, tubular maturation, and the differentiation of FOXD1+ stromal cells into smooth muscle, renin, and mesangial cells. (Gerl et al., 2017) This was found to be regulated by the inhibition of PhD2 and PhD3. (Kobayashi et al., 2017) The fact that the organoids do not mature further and mesangial cells and renin cells are thought to be absent in this kidney organoid model (Yousef Yengej et al., 2020), could indicate impaired *in vitro* differentiation of FOXD1+ progenitor cells. Therefore, future research could investigate the role of HIF-2α nuclear translocation in this context.

*In vivo*, pericytes derived from FOXD1+ progenitors are known to show HIF-2α nuclear translocation that induces EPO production. However, in our organoids, HIF-2α nuclear translocation did not induce EPO mRNA transcription in either normoxia or hypoxia (Supplementary Figure S8). The fact that EPO was not transcribed in the organoids in normoxia and hypoxia could potentially be due to a fibrotic stromal population found in the organoids (Li et al., 2004; Jagannathan et al., 2016) and subsequent hyper-methylation of the EPO 5’ and HRE, inhibiting HIF2/HIFβ dimer binding, as found in adult fibrotic kidneys (Shih et al., 2018). Future research could clarify mechanistically, if nuclear HIF-2α is actually leading to target gene transcriptions or if this is inhibited, consequently being one reason for limited organoid maturation. (Gerl et al., 2017)

As with HIF-2α expression, we found differences in the expression of VEGF-A in our kidney organoids compared to fetal human developing kidneys. Comparing the normoxic and hypoxic organoid culture, a decrease in VEGF-A protein expression could be seen. VEGF-A, being one of the most important angiogenic factors, is known for its function in nephrogenesis to induce blood vessel formation in glomeruli through branching angiogenesis and consequently to induce maturation of glomerular cells. (Kim et al., 2002) In fetal human developing kidneys, VEGF-A is expressed in the epithelial cells in the s-shaped body and collecting duct. (Tufró, 2000) Later in nephrogenesis, VEGF-A uptake by convoluted tubules has been observed. (Bevan et al., 2008) In the organoids, VEGF-A was localized on the apical side of LTL+ proximal tubules, co-expressing the LoH marker SLC12A1. We rarely detected expression in podocytes, which is needed to initiate the formation of glomerular capillaries. (Eremina and Quaggin, 2004) Consequently, glomeruli containing endothelial cells is a rare event in the organoids. The amount of VEGF-A expression differed between differentiations. However, VEGF-A was reliably less expressed in hypoxia. The location of VEGF-A at the apical side of SCL12A1+ tubules remained the same in hypoxia (Figure 4 A–B, D). We hypothesized that there could be differences in the VEGF-A isoform and transcript variant expression, which could remain hidden by targeting three isoforms with the same antibody as performed in Figures 4A,B. *In vivo*, podocytes are the main source of VEGF-A and synthesize three VEGF-A isoforms (VEGF-A-121, VEGF-A-165, VEGF-A-189) by alternative mRNA splicing. (Guan et al., 2006)

VEGF-A variants were differentially expressed in the organoids in hypoxia (Figures 5A–C), consistent with previous studies. (Farina et al., 2020) *VEGF-A189* was significantly upregulated in organoids cultured in hypoxia, which is associated with microvessel formation (Li et al., 2004; Jagannathan et al., 2016) and enhanced migration of endothelial cells (Dahan et al., 2021). Being positively charged in some domains, encoded for by exons 6a, 6b and 7, VEGF-A189 binds negatively charged extracellular matrix and heparan sulphate proteoglycans on cell surfaces, remaining spatially localized and becomes biologically active upon mobilization by heparinase. (Cébe Suarez et al., 2006; Roodink et al., 2006) This allows the attraction of vessels into hypoxic tissue. Enhanced cell migration is also confirmed in a variety of cell lines cultured at 0.5% O_2_ and modified to overexpress VEGF-A189. (Salton et al., 2014). In contrast to VEGF-189, VEGF-A121 lacks both exon 6 and 7 and is therefore a readily diffusible, active isoform. Hypoxia-induced pro-angiogenic VEGF-A gene alternative splicing is also known for the VEGF-A121 and VEGF-A165 variants (Farina et al., 2020). Their upregulation in the hypoxic culture could indicate an overall higher availability of the three isoforms throughout the organoid. While we could not prove the localization of these isoforms in the organoids due to unavailability of isoform-specific antibodies, we did find an improved patterning of the endothelial network in hypoxia compared to normoxia. Microvessels were homogenously sprouting (Figures 6A–C) with larger vessel length (Figure 6F) and larger connectivity in 3D (Supplementary video 1 and 2) in hypoxia. In normoxia, there was comparatively less connectivity, hetereogenous vessel morphologies and planar growth.

The VEGF-A165b isoform was downregulated in hypoxia compared to normoxia (Figure 4E). VEGF-A165b is a low efficacy agonist, binding VEGFR2 with a stronger affinity and thereby reducing binding of VEGF-A165, resulting in strongly decreased signal transduction via the VEGFR2 receptor. (Peach et al., 2018; Mamer et al., 2020) VEGF-A165b is upregulated in quiescent vessels and in adult kidneys (Li et al., 2004; Jagannathan et al., 2016), inhibiting endothelial cell migration (Li et al., 2004; Jagannathan et al., 2016), and is downregulated in nephrogenesis during capillary loop formation (Bevan et al., 2008; Stolz and Sims-Lucas, 2015). Downregulation of the VEGF-A165b isoform in hypoxia at day 7 + 25 could indicate increased binding of VEGF-A165 to the VEGFR2 receptor, enhanced signal transduction and consequently the initiation of angiogenesis. Earlier research confirms the phosphorylation of SRSF1 splice factor by hypoxia targeting the exon 8a proximal splice site, leading to the expression of angiogenic VEGF-A isoforms. (Farina et al., 2020) While the increased vessel sprouting observed in the hypoxia-cultured organoids is an indication of angiogenesis (Tahergorabi and Khazaei, 2012) (Figures 6A–C), more research is needed to prove causality with the downregulated VEGF-A165b isoform. Furthermore, to our knowledge, alternative splicing of VEGF-A in non-pathological developmental angiogenesis is insufficiently studied and would be highly valuable in the context of organoid vascularization and maturation. Finally, *in vivo* glomerular maturation is VEGF-A dose dependent, however, it is only hypothesized that the antiangiogenic isoforms have a dose dependent effect in glomerulogenesis as well. (Bevan et al., 2008).

The results of our study show that modulation of the oxygen concentrations in kidney organoid culture can improve the patterning of endothelial cells and therefore is potentially a relevant factor to integrate in regular organoid culture. Sprouting and interconnected vessels in hypoxia indicated the activation of angiogenesis, which is important for further nephron maturation. Future research is, however, needed to confirm the mechanism by which this enhanced patterning of the endothelial network takes place and to elucidate the roles of the different VEGF-A isoforms. This understanding will help to further enhance the endothelial network in kidney organoids and can potentially be applied to other systems as well. An important challenge in this context will be to understand the batch variations in VEGFA expression and number of endothelial cells in the organoids. Furthermore, we hypothesize that a culture in a hypoxic chamber could be a more controlled environment to study the effects of a hypoxic culture on organoid development. This setup would allow medium changes at the desired hypoxia instead of at ambient oxygen concentrations and would consequently avoid repeated reoxygenation of the organoids. Future research could investigate the effects of hypoxia on nephrons beyond structural development and cell type specific marker expression, such as mitochondrial functionality and cell type specific changes in metabolism. Ideal would be a comparison of the transcriptome of fetal human kidneys with organoids in different oxygen concentrations. Finally, it remains to be determined how VEGF-A expression by podocytes can be increased to allow vascularization and maturation of glomeruli.

## Conclusion

In conclusion, kidney organoid culture in physiological hypoxia induced the formation of a homogenous and interconnected endothelial network, while maintaining renal cell types and their spatial organization. We found that VEGF-A189, VEGF-A165 and VEGF-A121 mRNA is upregulated in hypoxia. At day 7+25 VEGF-A165 is no longer upregulated. Protein expression analysis of the antiangiogenic VEGF-A165b isoform confirmed significant downregulation in hypoxia at day 7+25, being a potential reason for the enhanced endothelial sprouting. While further vessel maturation, i.e., tube formation and glomerular vascularization, are still unresolved, we believe that culture in physiological hypoxia is an important driving force for organoid vascularization and is translatable to other organoid models in a model-specific manner.

## Data Availability

All data generated for this article, including high resolution Supplementary Videos, was made publicly available for download from https://doi.org/10.34894/KMRJPD.

## References

[B1] Abhinand C. S. Raju R. Soumya S. J. Arya P. S. Sudhakaran P. R. (2016). VEGF-A/VEGFR2 Signaling Network in Endothelial Cells Relevant to Angiogenesis. J. Cell Commun. Signal. 10 (4), 347–354. 10.1007/s12079-016-0352-8 27619687 PMC5143324

[B2] Bekhite M. M. Finkensieper A. Rebhan J. Huse S. Schultze-Mosgau S. Figulla H.-R. (2014). Hypoxia, Leptin, and Vascular Endothelial Growth Factor Stimulate Vascular Endothelial Cell Differentiation of Human Adipose Tissue-Derived Stem Cells. Stem Cells Dev. 23 (4), 333–351. 10.1089/scd.2013.0268 24134622

[B3] Bernhardt W. M. Schmitt R. Rosenberger C. Münchenhagen P. M. Gröne H.-J. Frei U. (2006). Expression of Hypoxia-Inducible Transcription Factors in Developing Human and Rat Kidneys. Kidney Int. 69 (1), 114–122. 10.1038/sj.ki.5000062 16374431

[B4] Bevan H. S. van den Akker N. M. S. Qiu Y. Polman J. A. E. Foster R. R. Yem J. (2008). The Alternatively Spliced Anti-Angiogenic Family of VEGF Isoforms VEGFxxxb in Human Kidney Development. Nephron Physiol. 110 (4), p57–p67. 10.1159/000177614 19039247 PMC2635558

[B5] Brueckl C. Kaestle S. Kerem A. Habazettl H. Krombach F. Kuppe H. (2006). Hyperoxia-Induced Reactive Oxygen Species Formation in Pulmonary Capillary Endothelial CellsIn Situ. Am. J. Respir. Cell Mol. Biol. 34 (4), 453–463. 10.1165/rcmb.2005-0223oc 16357365

[B6] Buchholz B. Schley G. Eckardt K.-U. (2016). The Impact of Hypoxia on Nephrogenesis. Curr. Opin. Nephrol. Hypertens. 25 (3), 180–186. 10.1097/mnh.0000000000000211 27023836

[B7] Cébe Suarez S. Pieren M. Cariolato L. Arn S. Hoffmann U. Bogucki A. (2006). A VEGF-A Splice Variant Defective for Heparan Sulfate and Neuropilin-1 Binding Shows Attenuated Signaling Through VEGFR-2. Cell. Mol. Life Sci. 63 (17), 2067–2077. 10.1007/s00018-006-6254-9 16909199 PMC11136335

[B8] Dahan S. Sharma A. Cohen K. Baker M. Taqatqa N. Bentata M. (2021). VEGFA's Distal Enhancer Regulates its Alternative Splicing in CML. Nar Cancer 3 (3), zcab029. 10.1093/narcan/zcab029 34316716 PMC8276762

[B9] Ding B. Sun G. Liu S. Peng E. Wan M. Chen L. (2020). Three-Dimensional Renal Organoids from Whole Kidney Cells: Generation, Optimization, and Potential Application in Nephrotoxicology *In Vitro* . Cell Transpl. 29, 963689719897066. 10.1177/0963689719897066 PMC7504083 32166969

[B10] Eremina V. Quaggin S. E. (2004). The Role of VEGF-A in Glomerular Development and Function. Curr. Opin. Nephrol. Hypertens. 13 (1), 9–15. 10.1097/00041552-200401000-00002 15090854

[B11] Fajersztajn L. Veras M. M. (2017). Hypoxia: From Placental Development to Fetal Programming. Birth Defects Res. 109 (17), 1377–1385. 10.1002/bdr2.1142 29105382

[B12] Farina A. R. Cappabianca L. Sebastiano M. Zelli V. Guadagni S. Mackay A. R. (2020). Hypoxia-Induced Alternative Splicing: The 11th Hallmark of Cancer. J. Exp. Clin. Cancer Res. 39 (1), 110. 10.1186/s13046-020-01616-9 32536347 PMC7294618

[B13] Fischer B. Bavister B. D. (1993). Oxygen Tension in the Oviduct and Uterus of Rhesus Monkeys, Hamsters and Rabbits. Reproduction 99 (2), 673–679. 10.1530/jrf.0.0990673 8107053

[B14] Freeburg P. B. Abrahamson D. R. (2003). Hypoxia-inducible Factors and Kidney Vascular Development. J. Am. Soc. Nephrol. 14 (11), 2723–2730. 10.1097/01.asn.0000092794.37534.01 14569081

[B15] Freeburg P. B. Robert B. St. John P. L. Abrahamson D. R. (2003). Podocyte Expression of Hypoxia-Inducible Factor (HIF)-1 and HIF-2 During Glomerular Development. J. Am. Soc. Nephrol. 14 (4), 927–938. 10.1097/01.asn.0000059308.82322.4f 12660327

[B16] Gerl K. Steppan D. Fuchs M. Wagner C. Willam C. Kurtz A. (2017). Activation of Hypoxia Signaling in Stromal Progenitors Impairs Kidney Development. Am. J. Pathology 187 (7), 1496–1511. 10.1016/j.ajpath.2017.03.014 28527294

[B17] Gerosa C. Fanni D. Faa A. Van Eyken P. Ravarino A. Fanos V. (2017). Low Vascularization of the Nephrogenic Zone of the Fetal Kidney Suggests a Major Role for Hypoxia in Human Nephrogenesis. Int. Urol. Nephrol. 49 (9), 1621–1625. 10.1007/s11255-017-1630-y 28573487

[B18] Geuens T. Ruiter F. A. A. Schumacher A. Morgan F. L. C. Rademakers T. Wiersma L. E. (2021). Thiol-ene Cross-Linked Alginate Hydrogel Encapsulation Modulates the Extracellular Matrix of Kidney Organoids by Reducing Abnormal Type 1a1 Collagen Deposition. Biomaterials 275, 120976. 10.1016/j.biomaterials.2021.120976 34198162

[B19] Grobstein C. (1956). Trans-Filter Induction of Tubules in Mouse Metanephrogenic Mesenchyme. Exp. Cell Res. 10 (2), 424–440. 10.1016/0014-4827(56)90016-7 13317909

[B20] Guan F. Villegas G. Teichman J. Mundel P. Tufro A. (2006). Autocrine VEGF-A System in Podocytes Regulates Podocin and its Interaction with CD2AP. Am. J. Physiology-Renal Physiology 291 (2), F422–F428. 10.1152/ajprenal.00448.2005 16597608

[B21] Gupta N. Dilmen E. Morizane R. (2021). 3D Kidney Organoids for Bench-To-Bedside Translation. J. Mol. Med. 99 (4), 477–487. 10.1007/s00109-020-01983-y 33034708 PMC8026465

[B22] Haddad J. J. Harb H. L. (2005). Cytokines and the Regulation of Hypoxia-Inducible Factor (HIF)-1α. Int. Immunopharmacol. 5 (3), 461–483. 10.1016/j.intimp.2004.11.009 15683844

[B23] Han Y. Kuang S.-Z. Gomer A. Ramirez-Bergeron D. L. (2010). Hypoxia Influences the Vascular Expansion and Differentiation of Embryonic Stem Cell Cultures Through the Temporal Expression of Vascular Endothelial Growth Factor Receptors in an ARNT-Dependent Manner. Stem Cells 28 (4), 799–809. 10.1002/stem.316 20135683 PMC2989499

[B24] Hemker S. L. Sims-Lucas S. Ho J. (2016). Role of Hypoxia During Nephrogenesis. Pediatr. Nephrol. 31 (10), 1571–1577. 10.1007/s00467-016-3333-5 26872484 PMC4982845

[B25] Jagannathan L. Cuddapah S. Costa M. (2016). Oxidative Stress Under Ambient and Physiological Oxygen Tension in Tissue Culture. Curr. Pharmacol. Rep. 2 (2), 64–72. 10.1007/s40495-016-0050-5 27034917 PMC4809260

[B26] Keeley T. P. Mann G. E. (2019). Defining Physiological Normoxia for Improved Translation of Cell Physiology to Animal Models and Humans. Physiol. Rev. 99 (1), 161–234. 10.1152/physrev.00041.2017 30354965

[B27] Kim B.-S. Chen J. Weinstein T. Noiri E. Goligorsky M. S. (2002). VEGF Expression in Hypoxia and Hyperglycemia: Reciprocal Effect on Branching Angiogenesis in Epithelial-Endothelial Co-Cultures. J. Am. Soc. Nephrol. 13 (8), 2027–2036. 10.1097/01.asn.0000024436.00520.d8 12138133

[B28] Klingberg A. Hasenberg A. Ludwig-Portugall I. Medyukhina A. Männ L. Brenzel A. (2017). Fully Automated Evaluation of Total Glomerular Number and Capillary Tuft Size in Nephritic Kidneys Using Lightsheet Microscopy. J. Am. Soc. Nephrol. 28 (2), 452–459. 10.1681/asn.2016020232 27487796 PMC5280021

[B29] Kobayashi H. Liu J. Urrutia A. A. Burmakin M. Ishii K. Rajan M. (2017). Hypoxia-Inducible Factor Prolyl-4-Hydroxylation in FOXD1 Lineage Cells Is Essential for Normal Kidney Development. Kidney Int. 92 (6), 1370–1383. 10.1016/j.kint.2017.06.015 28847650 PMC5696043

[B30] Lee Y. M. Jeong C.-H. Koo S.-Y. Son M. J. Song H. S. Bae S.-K. (2001). Determination of Hypoxic Region by Hypoxia Marker in Developing Mouse Embryos *in Vivo*: A Possible Signal for Vessel Development. Dev. Dyn. 220 (2), 175–186. 10.1002/1097-0177(20010201)220:2<175::aid-dvdy1101>3.0.co;2-f 11169851

[B31] Li J. Gao X. Qian M. Eaton J. W. (2004). Mitochondrial Metabolism Underlies Hyperoxic Cell Damage. Free Radic. Biol. Med. 36 (11), 1460–1470. 10.1016/j.freeradbiomed.2004.03.005 15135183

[B32] Loughna S. Yuan H.-T. Woolf A. S. (1998). Effects of Oxygen on Vascular Patterning in *Tie1/Lac* ZMetanephric Kidneys in Vitro. Biochem. Biophysical Res. Commun. 247 (2), 361–366. 10.1006/bbrc.1998.8768 9642132

[B33] Ma T. Grayson W. L. Fröhlich M. Vunjak-Novakovic G. (2009). Hypoxia and Stem Cell-Based Engineering of Mesenchymal Tissues. Biotechnol. Prog. 25 (1), 32–42. 10.1002/btpr.128 19198002 PMC2771546

[B34] Mamer S. B. Wittenkeller A. Imoukhuede P. I. (2020). VEGF-A Splice Variants Bind VEGFRs with Differential Affinities. Sci. Rep. 10 (1), 14413. 10.1038/s41598-020-71484-y 32879419 PMC7468149

[B35] Michiels C. Arnould T. Remacle J. (2000). Endothelial Cell Responses to Hypoxia: Initiation of a Cascade of Cellular Interactions. Biochimica Biophysica Acta (BBA) - Mol. Cell Res. 1497 (1), 1–10. 10.1016/s0167-4889(00)00041-0 10838154

[B36] Nishinakamura R. (2019). Human Kidney Organoids: Progress and Remaining Challenges. Nat. Rev. Nephrol. 15 (10), 613–624. 10.1038/s41581-019-0176-x 31383997

[B37] Okkelman I. A. Foley T. Papkovsky D. B. Dmitriev R. I. (2017). Live Cell Imaging of Mouse Intestinal Organoids Reveals Heterogeneity in Their Oxygenation. Biomaterials 146, 86–96. 10.1016/j.biomaterials.2017.08.043 28898760

[B38] Otrock Z. Mahfouz R. Makarem J. Shamseddine A. (2007). Understanding the Biology of Angiogenesis: Review of the Most Important Molecular Mechanisms. Blood Cells, Mol. Dis. 39 (2), 212–220. 10.1016/j.bcmd.2007.04.001 17553709

[B39] Peach C. Mignone V. Arruda M. Alcobia D. Hill S. Kilpatrick L. (2018). Molecular Pharmacology of VEGF-A Isoforms: Binding and Signalling at VEGFR2. Ijms 19 (4), 1264. 10.3390/ijms19041264 PMC5979509 29690653

[B40] Place T. L. Domann F. E. Case A. J. (2017). Limitations of Oxygen Delivery to Cells in Culture: An Underappreciated Problem in Basic and Translational Research. Free Radic. Biol. Med. 113, 311–322. 10.1016/j.freeradbiomed.2017.10.003 29032224 PMC5699948

[B41] Podkalicka P. Stępniewski J. Mucha O. Kachamakova-Trojanowska N. Dulak J. Łoboda A. (2020). Hypoxia as a Driving Force of Pluripotent Stem Cell Reprogramming and Differentiation to Endothelial Cells. Biomolecules 10 (12). 10.3390/biom10121614 PMC7759989 33260307

[B42] Prado-Lopez S. Conesa A. Armiñán A. Martínez-Losa M. Escobedo-Lucea C. Gandia C. (2010). Hypoxia Promotes Efficient Differentiation of Human Embryonic Stem Cells to Functional Endothelium. Stem Cells 28 (3), 407–418. 10.1002/stem.295 20049902

[B43] Roodink I. van der Laak J. Kusters B. Wesseling P. Verrijp K. de Waal R. (2006). Development of the Tumor Vascular Bed in Response to Hypoxia-Induced VEGF-A Differs from that in Tumors with Constitutive VEGF-A Expression. Int. J. Cancer 119 (9), 2054–2062. 10.1002/ijc.22072 16804907

[B44] Rossi G. Manfrin A. Lutolf M. P. (2018). Progress and Potential in Organoid Research. Nat. Rev. Genet. 19 (11), 671–687. 10.1038/s41576-018-0051-9 30228295

[B45] Salomon C. Ryan J. Sobrevia L. Kobayashi M. Ashman K. Mitchell M. (2013). Exosomal Signaling During Hypoxia Mediates Microvascular Endothelial Cell Migration and Vasculogenesis. PLoS One 8 (7), e68451. 10.1371/journal.pone.0068451 23861904 PMC3704530

[B46] Salton M. Voss T. C. Misteli T. (2014). Identification by High-Throughput Imaging of the Histone Methyltransferase EHMT2 as an Epigenetic Regulator of VEGFA Alternative Splicing. Nucleic Acids Res. 42 (22), 13662–13673. 10.1093/nar/gku1226 25414343 PMC4267647

[B47] Schindelin J. Arganda-Carreras I. Frise E. Kaynig V. Longair M. Pietzsch T. (2012). Fiji: An Open-Source Platform for Biological-Image Analysis. Nat. Methods 9 (7), 676–682. 10.1038/nmeth.2019 22743772 PMC3855844

[B48] Schneider C. A. Rasband W. S. Eliceiri K. W. (2012). NIH Image to ImageJ: 25 Years of Image Analysis. Nat. Methods 9 (7), 671–675. 10.1038/nmeth.2089 22930834 PMC5554542

[B49] Schumacher A. Rookmaaker M. B. Joles J. A. Kramann R. Nguyen T. Q. van Griensven M. (2021). Defining the Variety of Cell Types in Developing and Adult Human Kidneys by Single-Cell RNA Sequencing. NPJ Regen. Med. 6 (1), 45. 10.1038/s41536-021-00156-w 34381054 PMC8357940

[B50] Shang T. Li S. Zhang Y. Lu L. Cui L. Guo F. F. (2019). Hypoxia Promotes Differentiation of Adipose-Derived Stem Cells into Endothelial Cells Through Demethylation of EphrinB2. Stem Cell Res. Ther. 10 (1), 133. 10.1186/s13287-019-1233-x 31109374 PMC6528245

[B51] Shih H.-M. Wu C.-J. Lin S.-L. (2018). Physiology and Pathophysiology of Renal Erythropoietin-Producing Cells. J. Formos. Med. Assoc. 117 (11), 955–963. 10.1016/j.jfma.2018.03.017 29655605

[B52] Stolz D. B. Sims-Lucas S. (2015). Unwrapping the Origins and Roles of the Renal Endothelium. Pediatr. Nephrol. 30 (6), 865–872. 10.1007/s00467-014-2798-3 24633402 PMC4164630

[B53] Tahergorabi Z. Khazaei M. (2012). A Review on Angiogenesis and its Assays. Iran. J. Basic Med. Sci. 15 (6), 1110–1126. 23653839 PMC3646220

[B54] Takasato M. Er P. X. Chiu H. S. Little M. H. (2016). Generation of Kidney Organoids from Human Pluripotent Stem Cells. Nat. Protoc. 11 (9), 1681–1692. 10.1038/nprot.2016.098 27560173 PMC5113819

[B55] Takasato M. Er P. X. Chiu H. S. Maier B. Baillie G. J. Ferguson C. (2015). Kidney Organoids from Human iPS Cells Contain Multiple Lineages and Model Human Nephrogenesis. Nature 526 (7574), 564–568. 10.1038/nature15695 26444236

[B56] Takasato M. Wymeersch F. J. (2020). Challenges to Future Regenerative Applications Using Kidney Organoids. Curr. Opin. Biomed. Eng. 13, 144–151. 10.1016/j.cobme.2020.03.003

[B57] Tufró A. (2000). VEGF Spatially Directs Angiogenesis During Metanephric Development *in Vitro* . Dev. Biol. 227 (2), 558–566. 10.1006/dbio.2000.9845 11071774

[B58] Tufro-McReddie A. Norwood V. F. Aylor K. W. Botkin S. J. Carey R. M. Gomez R. A. (1997). Oxygen Regulates Vascular Endothelial Growth Factor-Mediated Vasculogenesis and Tubulogenesis. Dev. Biol. 183 (2), 139–149. 10.1006/dbio.1997.8513 9126290

[B59] van den Berg C. W. Ritsma L. Avramut M. C. Wiersma L. E. van den Berg B. M. Leuning D. G. (2018). Renal Subcapsular Transplantation of PSC-Derived Kidney Organoids Induces Neo-Vasculogenesis and Significant Glomerular and Tubular Maturation *In Vivo* . Stem Cell Rep. 10 (3), 751–765. 10.1016/j.stemcr.2018.01.041 PMC5918682 29503086

[B60] Vempati P. Popel A. S. Mac Gabhann F. (2014). Extracellular Regulation of VEGF: Isoforms, Proteolysis, and Vascular Patterning. Cytokine & growth factor Rev. 25 (1), 1–19. 10.1016/j.cytogfr.2013.11.002 24332926 PMC3977708

[B61] Wörsdörfer P. Ergün S. (2021). The Impact of Oxygen Availability and Multilineage Communication on Organoid Maturation. Antioxid. Redox Signal 35 (3), 217–233. 10.1089/ars.2020.8195 33334234

[B62] Xie F. Xiao P. Chen D. Xu L. Zhang B. (2012). miRDeepFinder: A miRNA Analysis Tool for Deep Sequencing of Plant Small RNAs. Plant Mol. Biol. 80, 75–84. 10.1007/s11103-012-9885-2 22290409

[B63] Yousef Yengej F. A. Jansen J. Rookmaaker M. B. Verhaar M. C. Clevers H. (2020). Kidney Organoids and Tubuloids. Cells 9 (6), 1326. 10.3390/cells9061326 PMC7349753 32466429

[B64] Yuan C. Wang P. Zhu L. Dissanayaka W. L. Green D. W. Tong E. H. (2015). Coculture of Stem Cells from Apical Papilla and Human Umbilical Vein Endothelial Cell Under Hypoxia Increases the Formation of Three-Dimensional Vessel-Like Structures *in Vitro* . Tissue Eng. Part A 21 (5-6), 1163–1172. 10.1089/ten.TEA.2014.0058 25380198 PMC4356259

