# Supplementary material for: Enhanced Microvasculature Formation and Patterning in iPSC–Derived Kidney Organoids Cultured in Physiological Hypoxia
Source: Front Bioeng Biotechnol. 2022 Jun 13;10:860138. doi: 10.3389/fbioe.2022.860138 (PMC9240933; doi:10.3389/fbioe.2022.860138)
Supplement: Supplementary file 2 [file DataSheet1.pdf]

Supplementary Material

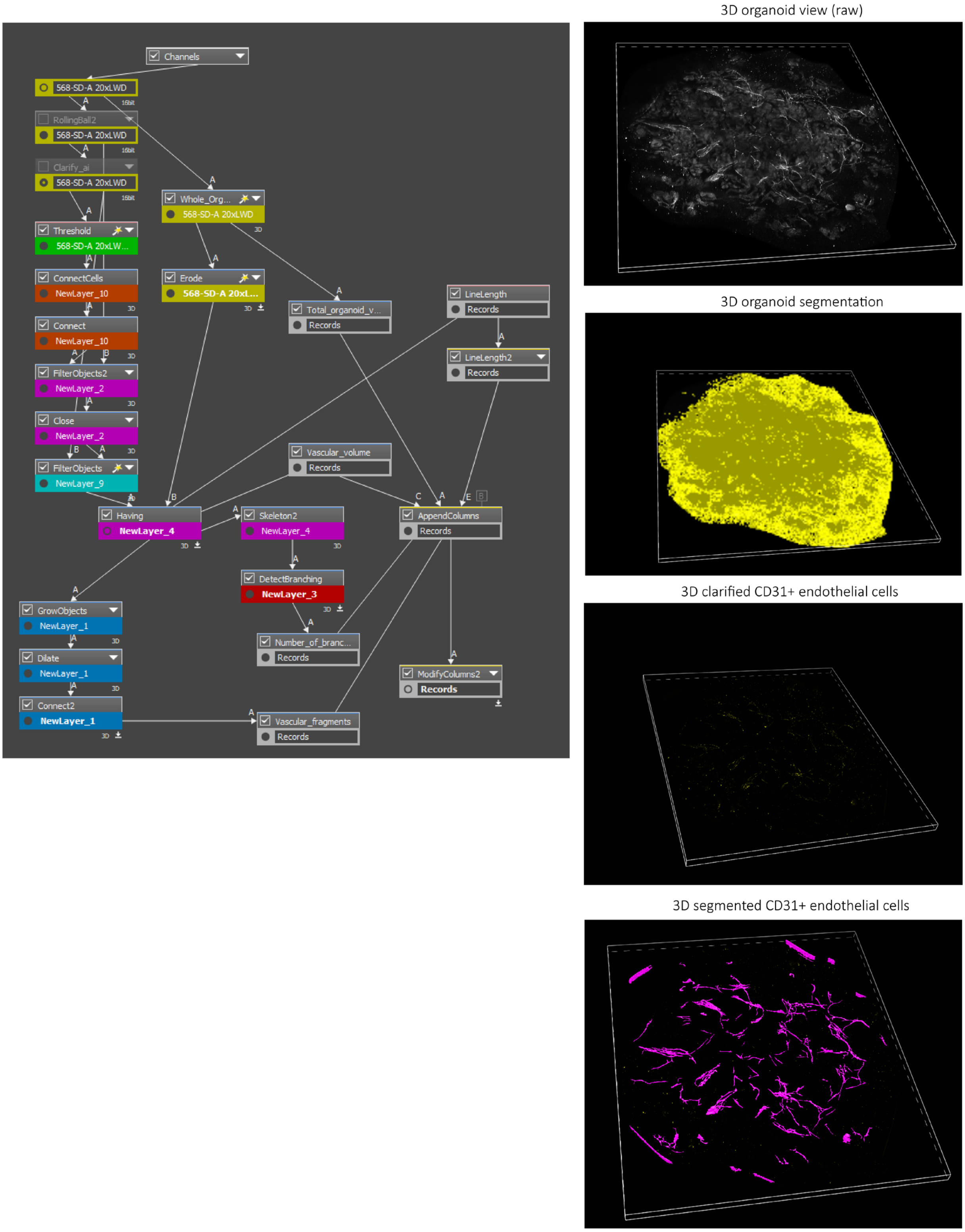

Supplementary figure 1: Segmentation and quantification pipeline of the endothelial network in 3D. Detailed description can be found in the methods section. Briefly, after clarifying the image, the CD31 signal was thresholded, and after conservative filtering, the vessels were skeletonized and segmented.

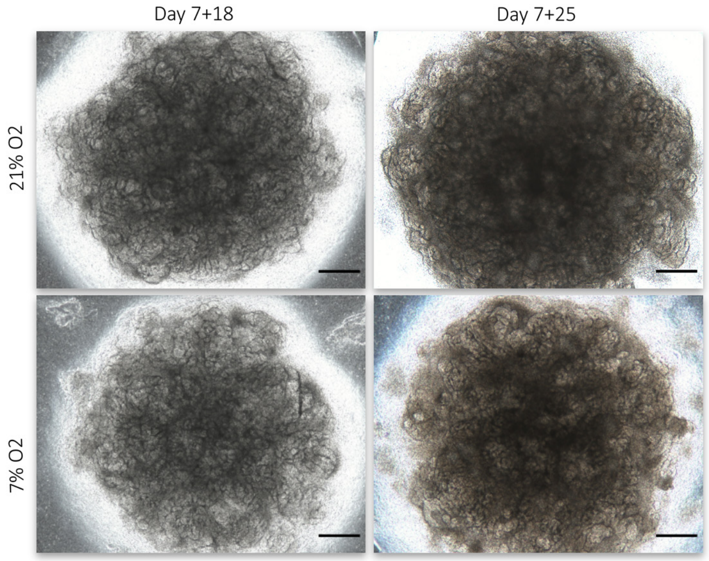

Supplementary figure 2: Brightfield images of organoids in normoxia and hypoxia. Kidney organoids in 21% O_2 and 7% O_2 develop similar morphologies. Scale bars: 1 mm

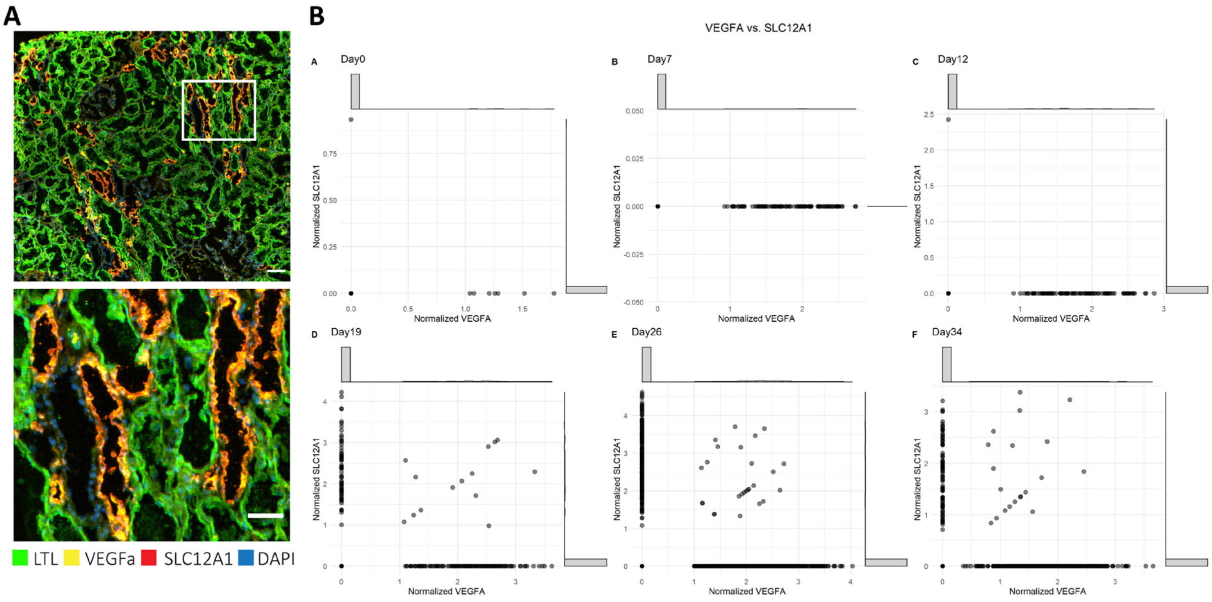

Supplementary figure 3: Vascular endothelial growth factor alpha (VEGF-A) marker validation on adult human kidney and in single cell RNA sequencing datasets of kidney organoids. VEGF-A is co-expressed with Solute carrier family 12 member 1 (SLC12A1) in both adult human kidney sections (A) and single cell RNA sequencing data (B) of Wu et al (2018). Scale bars: 50 µm

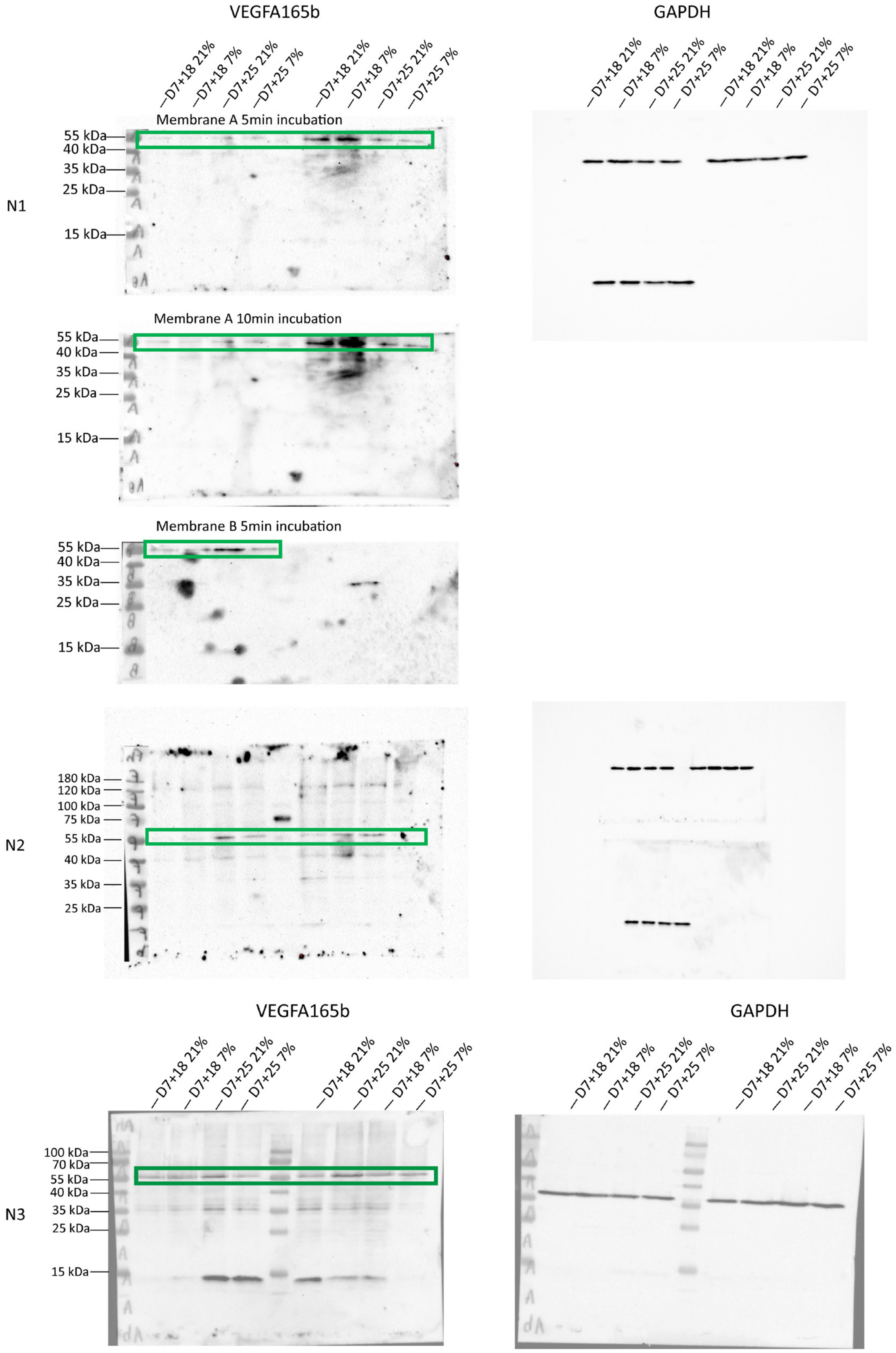

Supplementary figure 4: Raw blots of VEGF-A165b and GAPDH, indicating the downregulation of VEGF-165b in hypoxia compared to normoxia at D7+25.

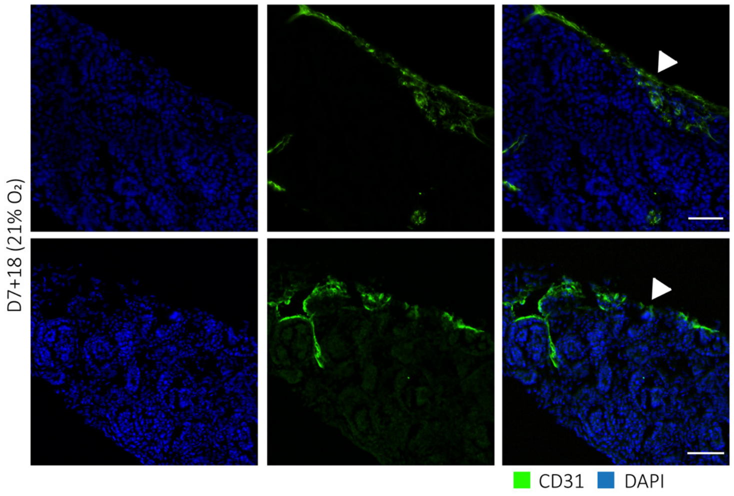

Supplementary figure 5: Localization of endothelial cells in kidney organoids. Immunofluorescence staining of D7+18 (21% O2) shows that endothelial cells are largely located on the surface of the organoids (arrow indicating the air-exposed surface) and only rarely infiltrate into the organoids or reside on the bottom and leaving them directly exposed to the hyperoxic incubator air. Scale bars: 50 µm (top row), 70 µm (bottom row).

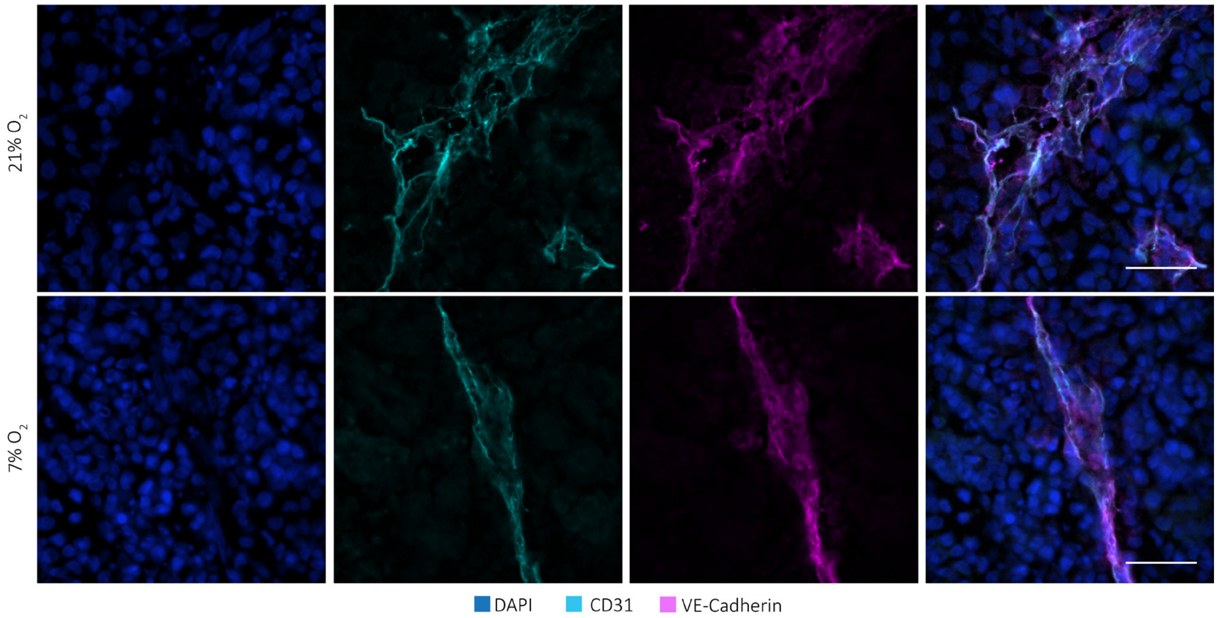

Supplementary figure 6: CD31 and VE-cadherin expression at day 7+25 at 21% and 7% O_2. Co-expression of CD31 and VE-cadherin indicates maintenance of the endothelial phenotype at day 7+25 in culture in both 21% and 7% O_2. Scale bar: 30 µm

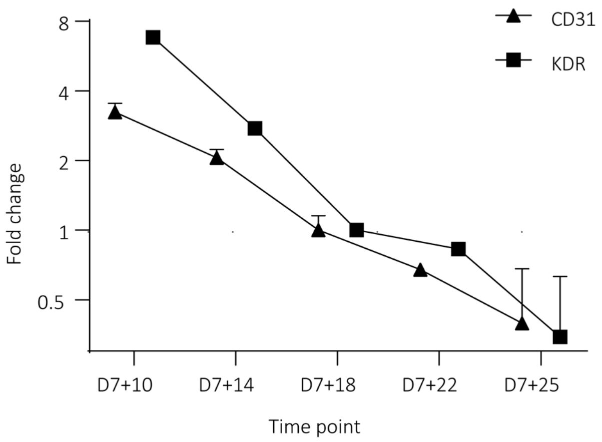

Supplementary figure 7: Gene expression of CD31 and KDR is downregulated over time in 21% O_2 indicating a diminishing endothelial network.

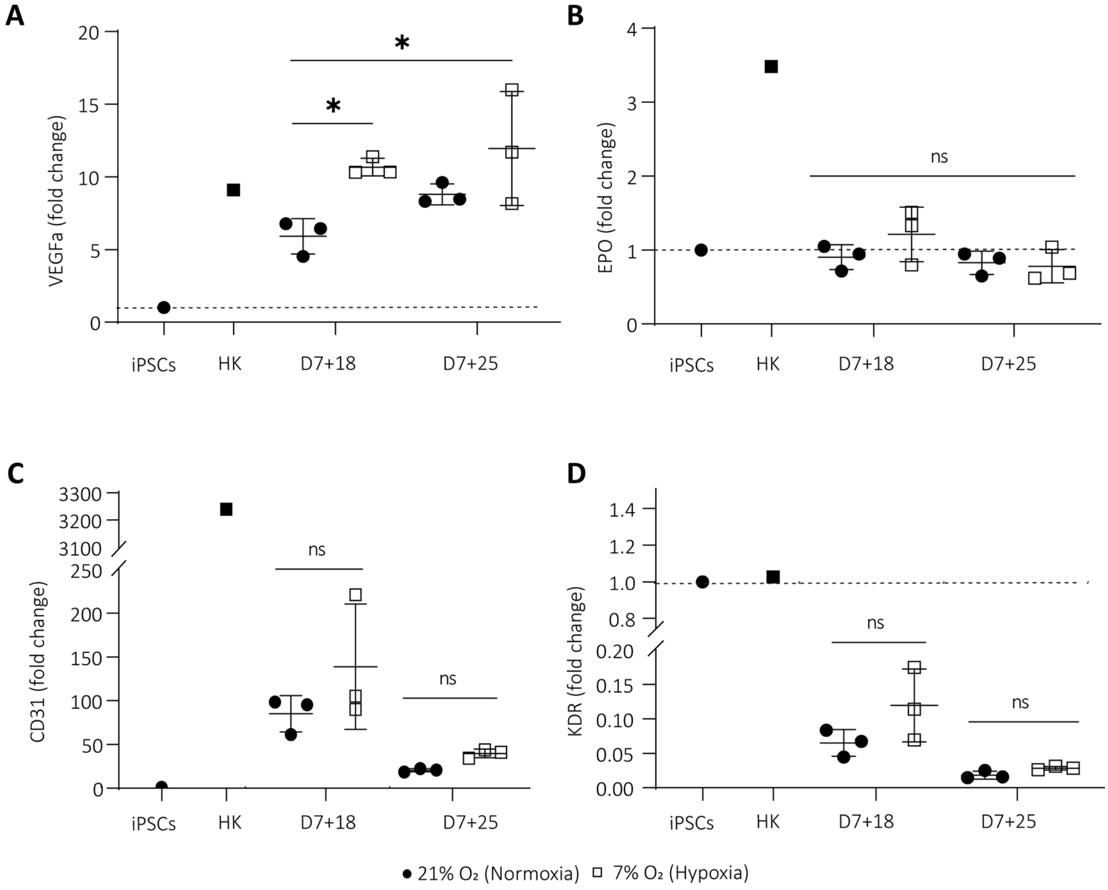

Supplementary figure 8: Gene expression of VEGF-A (all variants), EPO, CD31 and KDR in 21% O_2 and 7% O_2 of a single experiment. VEGF-A mRNA is significantly upregulated at 7% O_2 (A). There is no significant difference between 21% O_2 and 7% O_2 in EPO (B), CD31 (C) and KDR (D) gene expression. Human kidney and iPSCs were plotted as reference. (n=3, N=1)

Supplementary Tables

Supplementary Table 1: List of antibodies and stains used for immunofluorescence of cryosections and whole organoids.

Target	Host	Dilution	Supplier	Cat. no.
**Primary antibodies and stains**				
CD31	Sheep	1:300	R&D Systems	AF809
E-cadherin (ECAD)	Mouse	1:300	BD Bioscience	610182
HIF1α	Mouse	1:300	Abcam	ab16066
HIF2α	Mouse	1:250	Santa Cruz Biotechnology	sc-46691
Lotus tetragonolobus lectin (LTL), Biotinylated	N/A	1:300	Vector Laboratories	B-1325-2
MEIS 1/2/3	Mouse	1:300	Santa Cruz Biotechnology	SC-101850
Nephrin (NPHS1)	Sheep	1:300	R&D Systems	AF4269
SLC21A1	Rabbit	1:200	Sigma-Aldrich	HPAG14967
VEGF-A	Mouse	1:250	Santa Cruz Biotechnology	SC-7269
VEGF-A165b	Mouse	1:500	R&D Systems	MAB3045
WT1	Rabbit	1:500	Abcam	ab89901
**Secondary antibodies and stains**				
Anti-Mouse 488	Goat	1:400	Thermo Fisher Scientific	10696113
Anti-Mouse 568	Goat	1:400	Thermo Fisher Scientific	A-11031
Anti-Mouse 647	Goat	1:400	Thermo Fisher Scientific	A-21236
Anti-Rabbit 568	Donkey	1:400	Thermo Fisher Scientific	10617183
Anti-Sheep 488	Donkey	1:400	Thermo Fisher Scientific	10473982
Anti-Sheep 568	Donkey	1:400	Thermo Fisher Scientific	A-21099
Goat Anti-Mouse IgG (H + L)-HRP Conjugate	Mouse	1:3000	Bio-Rad	1706516
Streptavidin 647	N/A	1:400	Thermo Fisher Scientific	S21374

Supplementary Table 2: List of qPCR primers.

NCBI gene ID	Gene symbol	Forward primer (5' to 3')	Reverse primer (5' to 3')
283	ANG	CAAGGCCATCTGTGAAAACAAG	CAGGGGGAACCTCCATGTAG
2056	EPO	GACATAGTGGCCATGGATGAAG	CGAGGCCAAAGCAGATGAG
2597	GADPH[a]	CTGGGCTACACTGAGCACC	AAGTGGTCGTTGAGGGCAATG
3791	KDR	GGCCCAATAATCAGAGTGGCA	CCAGTGTCATTTCCGATCACTTT
5175	PECAM1	AACAGTGTTGACATGAAGAGCC	TGTAAAACAGCACGTCATCCTT
5692	PSMB4[b]	TGGCTCGTTTCCGCAACAT	GAAATCAGCGTAGTCGCCAG
7422	VEGF-A	AGGGCAGAATCATCACGAAGT	AGGGTCTCGATTGGATGGCA
7422	VEGF-A 121	AGGCCAGCACATAGGAGAGA	GCCTCGGCTTGTCACATTTTT
7422	VEGF-A 165	GAGCAAGACAAGAAAATCCC	CCTCGGCTTGTCACATCTG
7422	VEGF-A 189	TAAGTCCTGGAGCGTTCCCT	ACGCGAGTCTGTGTTTTTGC

[a] second housekeeping gene used to validate the results

[b] housekeeping gene used for analysis
